# The Chemistry of Zirconium/Carboxylate Clustering Process: Acidic Conditions to Promote Carboxylate-Unsaturated Octahedral Hexamers and Pentanuclear Species

**DOI:** 10.1021/acs.inorgchem.1c03466

**Published:** 2022-03-14

**Authors:** Jon Pascual-Colino, Beñat Artetxe, Garikoitz Beobide, Oscar Castillo, Maria Luz Fidalgo-Mayo, Ainhoa Isla-López, Antonio Luque, Sandra Mena-Gutiérrez, Sonia Pérez-Yáñez

**Affiliations:** † Departamento de Química Orgánica e Inorgánica, Facultad de Ciencia y Tecnología, Universidad del País Vasco/Euskal Herriko Unibertsitatea, UPV/EHU, Apartado 644, Bilbao E-48080, Spain; ‡ BCMaterials, Basque Center for Materials, Applications and Nanostructures, UPV/EHU Science Park, Leioa E-48940, Spain; § Departamento de Química Orgánica e Inorgánica, Facultad de Farmacia, Universidad del País Vasco/Euskal Herriko Unibertsitatea, UPV/EHU, Vitoria-Gasteiz E-01006, Spain

## Abstract

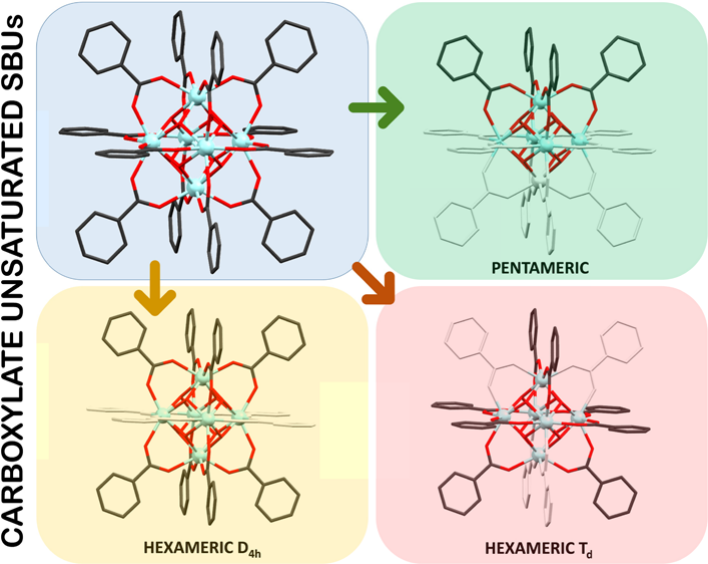

Clustering chemistry is a key point in the design and synthesis of the secondary building units that comprise metal–organic frameworks (MOFs) based on group IV metals. In this work, the first stages of the zirconium-carboxylate clustering process in alcohol/water mixtures are studied in detail using the monocarboxylic benzoic and hydroxybenzoic acids to avoid the polymerization. Mass spectroscopy measurements performed on the reactions revealed the presence of hexa- and pentanuclear species even at low pH values and also evidenced the acid–base nature and pH dependence of the transformation between both species. The control on the chemistry governing the equilibria between these species has allowed us to isolate six new compounds in the solid state. The single-crystal X-ray diffraction analysis revealed that they are closely related to the well-known [Zr_6_(O)_4_(OH)_4_(OOC)_12_] secondary building unit found in many MOFs by removing carboxylic ligands in the case of the hexameric species ([Zr_6_(O)_4_(OH)_4_(OOC)_8_(H_2_O)_8_]^4+^) or by additionally removing one of the metal centers in the case of the pentameric entities ([Zr_5_(O)_2_(OH)_6_(OOC)_4_(H_2_O)_11_(alcohol)]^6+^). Going in detail, the unsaturated hexameric clusters exhibit different dispositions of their eight carboxylate ligands in such a way that the remaining four carboxylate-free positions are arranged according to a square planar or tetrahedral symmetry. It should be highlighted that the pentameric complexes imply an unprecedented core nuclearity in zirconium clusters and thus their isolation provides a novel building block for the design of metal–organic materials.

## Abstract 2

Acidic synthesis conditions allow obtaining carboxylate-unsaturated zirconium octahedral hexamers and unprecedented pentanuclear species. Crystallography and mass spectroscopy evidences are provided.

## 1 Introduction

The interest in the design and preparation of discrete polynuclear metal–organic entities has resurfaced^1^ not only in the areas of classical magnetism^2^ and drug development^3,4^ but also in the discovery of new building units to design metal–organic frameworks (MOFs), which show endless applications based on the tailorability of their porosity.^5^ The key point for this fascinating diversity relies on the modular building up of their crystalline structure based on the combination of organic linkers and secondary building units (SBUs) consisting mainly of metal nodes or clusters.^6−8^ Until recently, novel topologies emerged basically from the change of the organic linkers, e.g., moving from ditopic to tritopic linkers. However, this approach has led to a never-ending increase of the complexity and cost of the bridging ligands employed to develop new MOFs.^9−12^

The other constituent, the SBUs, seems to be less explored, as usually the synthetic chemistry relies on the self-assembled metal-oxide-hydroxide polynuclear entities and they apparently show little variability under the conventional synthetic conditions employed for the preparation of these materials.^13,14^ This fact is evident when analyzing the reported structures for the zirconium/carboxylate MOF family, which are mostly based on the neutral [Zr_6_(O)_4_(OH)_4_(OOC)_12_] SBU. Although the resulting systems are both chemically and thermally robust mostly due to the strength of the Zr–O bond,^15,16^ the diversity of the porous features and topology relies entirely on the organic linker side. Regarding the zirconium/carboxylate entities, there are also some early works on discrete Zr_6_(O)_4_(OH)_4_ clusters using small monocarboxylates as capping agents.^17−20^ In this context, more recently, in situ pair distribution function (PDF) analysis confirmed the presence of the hexameric zirconium cluster in the metal salt precursor/DMF/HCl solution prior to the addition of the carboxylic organic ligand.^21^ As previously stated, the novel members of the zirconium MOF family rely on increasingly more complex and expensive polycarboxylic ligands. Therefore, there is a great interest in developing novel architectures based on low-cost aromatic polycarboxylic ligands by modifying the features of the SBUs.^22,23^ In this sense, a deep research work on the early stages of the formation of these polynuclear entities is required.^24^ Taking into account these premises, we have thoroughly analyzed the formation of discrete zirconium-oxide-hydroxide entities in alcoholic media using simple monocarboxylic benzoato and hydroxybenzoato ligands to avoid the polymerization that would hinder this kind of studies. A crucial stage of the setup of the Zr–O/OH polynuclear entities resides on the oxygen source from which these species emerge. In this sense, a precise control of the amount of water is crucial for the first steps of the formation of these entities.^25^ On the other hand, the acidity of the reaction media exerts a strong influence on the deprotonation of the coordinated water molecules to afford bridging hydroxide and oxide anions but also on the readiness of the carboxylic ligands to coordinate to the metal centers.^26^

**Figure 1:**
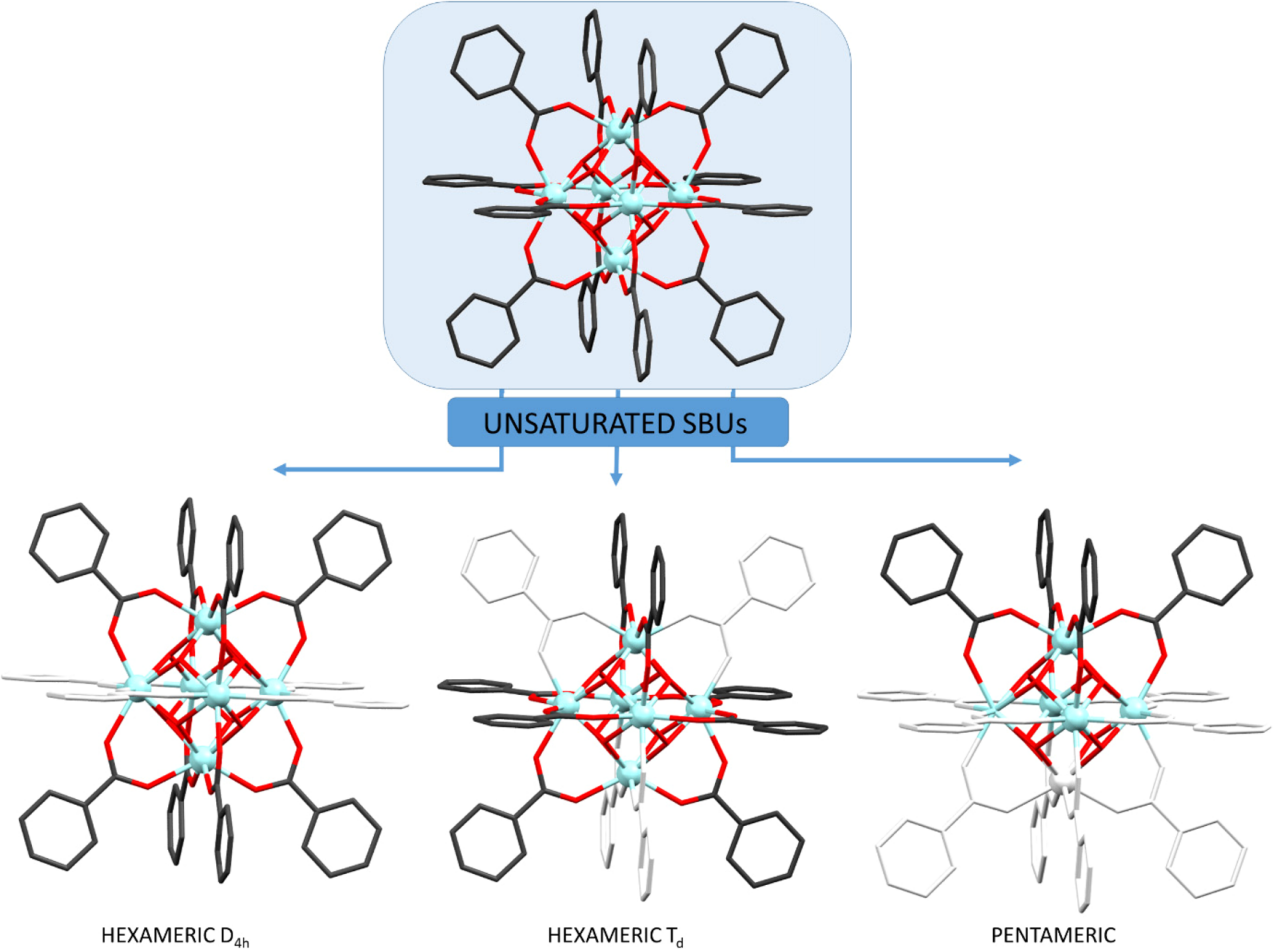
Schematic description of the unsaturated species obtained in this work and their relation with the well-known [Zr_6_(O)_4_(OH)_4_(OOC)_12_] fragment found in many MOFs (missing ligands are colored in light gray).

Herein, we report on several discrete zirconium entities ranging from the ubiquitous hexanuclear [Zr_6_(μ_3_-O)_4_(μ_3_-OH)_4_]^12+^ core obtained by capping some of the linking positions of the SBUs with the anionic forms of the selected monocarboxylic ligands ([Zr_6_(O)_4_(OH)_4_(L)_8_(H_2_O)_8_]^4+^ where L = benzoato in **1** and **2**, 2-hydroxybenzoato or salicylato in **3**, and 3-hydroxybenzoato in **4**) to a previously unknown pentanuclear [Zr_5_(μ_3_-O)_2_(μ_3_-OH)_2_(μ-OH)_4_]^10+^ core ([Zr_5_(O)_2_(OH)_6_(L)_4_(H_2_O)_11_(ROH)]^6+^ where L = benzoato; R = Et in **5**, Pr in **6**). Interestingly, in the case of the former octahedral-shaped hexanuclear entities, the coordination of the carboxylic ligands can be frozen in a cationic intermediate state in which only some of the available positions are occupied, leaving what can be called a carboxylate-unsaturated SBU (Figure 1).^27^ It is worth mentioning that zirconium-based MOFs are frequently carboxylate-defective (due to random linker vacancies or due to the restraints coming from the topology of the framework) but, in contrast to the compounds reported herein, the charge is balanced by the incorporation at these defective positions of smaller monocarboxylato ligands (formato and acetato) or by hydroxide anions.^28,29^ It will be also shown, how the noncovalent interactions coming from the hydroxyl-substituted positions direct the arrangement of the monocarboxylic ligands toward different symmetries regarding the unoccupied carboxylato positions: these have been placed in a square arrangement for benzoato (**1** and **2**) and 3-hydroxybenzoato (**4**) ligands and in a tetrahedral one for the 2-hydroxybenzoato (**3**) ligand.

## 2 Experimental Procedures

### 2.1 Chemicals

Zirconium(IV) chloride (ZrCl_4_, Sigma-Aldrich, anhydrous, for synthesis), benzoic acid (C_6_H_6_O_2_, Sigma-Aldrich, 99.5%), salicylic acid (C_4_H_6_O_3_, Sigma-Aldrich, 99%) 3-hydroxybenzoic acid (C_4_H_6_O_3_, Sigma-Aldrich, 99%), absolute ethanol (C_2_H_6_O, Scharlau), propanol (C_3_H_8_O, PanReac, HPLC grade). All the chemicals were of reagent grade and used as commercially obtained, except ethanol that was dehydrated using anhydrous CaSO_4_ in the synthesis of compounds **5** and **6**.

#### 2.1.1 Synthesis of Compounds **1**, **2**, and **5**

For the synthesis of these three compounds, a solution of 0.3870 g (1.66 mmol) of ZrCl_4_ dissolved in 4.8/0.2 mL of ethanol/water solution was added dropwise to 8 mL of ethanol solution containing 0.4054 g (3.32 mmol) of benzoic acid. The resulting colorless solution was basified dropwise with water until pH ≈ 0.0 (**2**), pH ≈ 0.5 (**1**), or left at pH < −0.2 (**5**). Reaction mixtures were left to evaporate at room temperature and colorless single crystals appeared after 3–7 days.

##### 2.1.1.1 [Zr_6_(μ_3_-O)_4_(μ_3_-OH)_4_(μ-OOCC_6_H_5_)_8_(H_2_O)_8_]Cl_4_·EtOH·35H_2_O (**1**)

Main IR features (cm^–1^; KBr pellets): 3370vs, 1600s, 1555s, 1525s, 1495s, 14290vs, 1305m, 1180s, 1155m, 1065s, 1025s, 935m, 840w, 720s, 660vs, 465m. ^1^H MA NMR δ (300 MHz): 7.2 ppm [aromatic H], 3.6 ppm [Zr-OH_2_]. ^13^C MAS NMR δ (300 MHz): 172 ppm [COOH], 132 ppm [C aromatic].

##### 2.1.1.2 [Zr_6_(μ_3_-O)_4_(μ_3_-OH)_4_(μ-OOCC_6_H_5_)_8_(H_2_O)_8_]Cl_4_·15H_2_O (**2**)

Main IR features (cm^–1^; KBr pellets): 3410vs, 1600s, 1555s, 1528vs, 1490s, 1420vs, 1300m, 1180s, 1155m, 1070s, 1020s, 930m, 840w, 720s, 653vs, 460m. ^1^H MAS NMR δ (300 MHz): 7.2 ppm [aromatic H], 3.6 ppm [Zr-OH_2_]. ^13^C MAS NMR δ (300 MHz): 172 ppm [COOH], 132 ppm [C aromatic].

##### 2.1.1.3 [Zr_5_(μ_3_-O)_2_(μ_3_-OH)_2_(μ-OH)_4_(μ-OOCC_6_H_5_)_4_(H_2_O)_11_(EtOH)]Cl_6_·2EtOH·10H_2_O (**5**)

Main IR features (cm^–1^; KBr pellets): 3420vs, 1622w, 1640w, 1600s, 1560m, 1531vs, 1490m, 1415vs, 1310m, 1175m, 1152w, 1070m, 1018m, 942m, 840w, 720s, 674s, 650m, 524w, 458m. ^1^H MAS NMR δ (300 MHz): 7.0 ppm [aromatic H], 4.3 ppm [Zr-OH_2_], 3.0 ppm [CH_2_], 0.4 ppm [CH_3_]. ^13^C MAS NMR δ (300 MHz): 172 ppm [COOH], 130 [C aromatic], 58 ppm [CH_2_], 17 ppm [CH_3_].

#### 2.1.2 Synthesis of Compound **6**

Compound **6** was prepared by mixing a solution of ZrCl_4_ (0.3870 g, 1.66 mmol) in 2.5/2.5 mL of ethanol/propanol mixture and benzoic acid (0.4054 g, 3.32 mmol) in 8 mL of propanol. The resulting colorless solution was left at pH < −0.2. Slow evaporation of the reaction mixture at room temperature yielded colorless single crystals 4 days later.

##### 2.1.2.1 [Zr_5_(μ_3_-O)_2_(μ_3_-OH)_2_(μ-OH)_4_(μ-OOCC_6_H_5_)_4_(H_2_O)_11_(PrOH)]Cl_6_·2PrOH·11H_2_O (**6**)

Main IR features (cm^–1^; KBr pellets): 3410vs, 1622w, 1640w, 1600s, 1560s, 1520vs, 1490s, 1410vs, 1306m, 1180m, 1155w, 1070m, 1020m, 940m, 840w, 720vs, 671s, 650m, 519w, 470m. ^1^H MAS NMR δ (300 MHz): 7.3 ppm [aromatic H], 4.5 ppm [Zr-OH_2_], 2.5 ppm [CH_2_], −0.5 ppm [CH_3_]. ^13^C MAS NMR δ (300 MHz): 172 ppm [COOH], 131 [C aromatic], 63 ppm [CH_2_], 22 ppm [CH_2_], 9 ppm [CH_3_].

#### 2.1.3 Synthesis of Compounds **3** and **4**

For the synthesis of these compounds, a solution of 0.3870 g (1.66 mmol) of ZrCl_4_ dissolved in 4.8/0.2 mL of ethanol/water solution was added dropwise to 8 mL of ethanol solution containing the corresponding ligand, 0.4586 g (3.32 mmol) of 2-hydroxybenzoic acid for compound **3** or 3-hydroxybenzoic acid for compound **4**. The resulting colorless solution was basified dropwise with water until pH ≈ 0.5 (**3**) or pH ≈ 1.0 (**4**). Reaction mixtures were left to evaporate at room temperature and colorless single crystals appeared after 3–7 days.

##### 2.1.3.1 [Zr_6_(μ_3_-O)_4_(μ_3_-OH)_4_(μ-OOCC_6_H_5_O)_8_(H_2_O)_8_]Cl_4_·28H_2_O (**3**)

Main IR features (cm^–1^; KBr pellets): 3340s, 1622s, 1586s, 1551s, 1484m, 1466s, 1395vs, 1311s, 1244vs, 1160s, 1144s, 1097m, 1026s, 951m, 808s, 755vs, 648m, 475w, 422w. ^1^H MAS NMR δ (300 MHz): 9.6 ppm [C-OH], 6.5 ppm [aromatic H], 2.96 ppm [Zr-OH_2_]. ^13^C MAS NMR δ (300 MHz): 173 ppm [COOH], 159 ppm [C-OH], 137 and 113 ppm [C aromatic].

##### 2.1.3.2 [Zr_6_(μ_3_-O)_4_(μ_3_-OH)_4_(μ-OOCC_6_H_5_O)_8_(H_2_O)_8_]Cl_4_·27H_2_O (**4**)

Main IR features (cm^–1^; KBr pellets): 3380s, 1608s, 1564s, 1533w, 1493w, 1448s, 1413s, 1302s, 1253s, 1231w, 1160m, 1120s, 1075s, 1000w, 942w, 795s, 764vs, 657vs, 457m. ^1^H MAS NMR δ (300 MHz): 7.1 ppm [aromatic H], 4.3 ppm [Zr-OH_2_]. ^13^C MAS NMR δ (300 MHz): 172 ppm [COOH], 154 ppm [C-OH], 132 and 120 ppm [C aromatic].

Regarding the measured pH values, if the calibration of the electrode is performed in aqueous buffers, but the measurement is performed in a different solvent, the measured pH requires to be subtracted with a correction constant: _s_^s^pH = _w_^s^pH – δ, where _s_^s^pH and _w_^s^pH would in this case correspond to the pH for solvent media and the measured pH, while δ is a correction constant. This constant depends of the solvent and it can be approached to −2.54 for ethanol. The pH values mentioned in this work have not been corrected and correspond to _w_^s^pH.^30,31^

### 2.2 Characterization

As the crystals of these compounds lose crystallinity upon their removal from the mother liquor, the purity of the samples was proved by FTIR (Fourier transform infrared spectroscopy, Table S2), TGA (thermogravimetric analysis, Table S3) and solid state NMR (nuclear magnetic resonance) spectroscopies, together with powder X-ray diffraction (PXRD) experiments performed over samples introduced in Lindemann tubes altogether with their mother liquors and using a Debye Scherrer instrument geometry. Lindemann capillary PXRD data were collected using a Rigaku SmartLab automatic diffractometer operating at 40 kV and 50 mA. The 2θ scans in transmission mode were obtained with parallel beam configuration (CBO), a capillary attachment head, an automatic attenuator, and a 1-D DteX250 detector. The diffraction data were collected in continuous rotation, from 3 to 65° step size of 0.01° at 0.5°/min scan speed. Routine PXRD measurements on filtered off samples were performed on a Philips X’PERT diffractometer (equipped with Cu-Kα radiation, λ = 1.5418 Å) over the range 5° < 2θ < 70° with a step size of 0.02°, a variable automatic divergence slit, and an acquisition time of 2.5 s per step at 293 K.

FTIR spectra of the samples (KBr pellet) were recorded at a resolution of 4 cm^–1^ in the 4000–500 cm^–1^ region using an FTIR 8400S Shimadzu spectrometer. ATR-FTIR spectra of the compounds while submerged in their mother liquids were obtained using an attenuated total reflectance (ATR) device equipped with a special concave head attached to an FTIR 8400S Shimadzu spectrometer.

Thermal analysis was performed on a METTLER TOLEDO TGA/SDTA851 thermal analyzer in a synthetic air (80% N_2_, 20% O_2_) flux of 50 cm^3^ min^–1^, from room temperature to 800 °C with a heating rate of 5 °C min^–1^ and about 10–20 mg of sample per run.

Solid state NMR measurements were performed on powder samples. High-resolution solid-state NMR spectra were recorded at 298 K on a Bruker Advance 400 WB spectrometer at 9.4 T, using 100.66 and 400.17 MHz resonance frequencies. The ^13^C experiments were performed with cross-polarization, high power decoupling, and magic angle spinning (MAS) configurations using a Bruker double-bearing probe head and 4 mm zirconia rotors driven by dry air. The MAS rates were 10 kHz. The Hartmann–Hahn conditions for ^13^C were matched using adamantane. The recycle delay was 5 s and the contact time was 2 ms. Chemical shifts were established using glycine (Gly) as an external standard (δ_CO of Gly_ = 176.5 ppm).

Electrospray ionization mass spectrometry (MS) analysis was conducted in an infusion of the reaction mixtures to a high-resolution mass spectrometer (Synapt G2 from Waters Cromatografia S.A., time of flight analyzer) at a flow rate of 20 μL/min by an electrospray ionization source in positive and negative modes. High resolution data were acquired in scan mode, using a mass range of 30–1200 u in resolution mode (FWHM ≈ 20,000) and a scan time of 0.1 s. The source temperature was set to 120 °C and the desolvation temperature to 350 °C. The capillary voltage was 2.5 kV (negative) and the cone voltage was 15 V. Nitrogen was used as the desolvation and cone gas at flow rates of 600 and 10 L/h, respectively. Before analysis, the mass spectrometer was calibrated with a sodium formate solution and a leucine enkephalin solution was used for the lock mass correction, monitoring the ions at a mass-to-charge ratio (*m*/*z*) of 556.2771. All of the acquired spectra were automatically corrected during acquisition based on the lock mass. Further details are available in the Supporting Information.

Single-crystal XRD data for structure determination were collected on Agilent Technologies Supernova diffractometers (λMοK_α_ = 0.71073 Å for **1**, **2**, **3**, **5** and CuK_α_ = 1.54184 Å for **4** and **6**). The data reduction was done with the CrysAlisPro program.^32^ Crystal structures were solved by direct methods using the SIR92^33^ and SHELXS^34^ programs and refined by full-matrix least-squares on *F*^2^ including all reflections (WINGX).^35,36^ Some crystal structures of some of the reported compounds show crystallographic disorder in the positions of some of the chloride anions and/or the aromatic ring of the carboxylic ligands. The disorder was modeled distributing the disordered atoms over two positions and fixing the sum of their occupation factors to one. The crystal structure of all the compounds revealed the presence of large channels in which the solvent molecules (water, ethanol, and propanol) are placed. The high disorder that solvent molecules present precluded their modeling and, as a consequence, the electron density at the voids of the crystal structure was subtracted from the reflection data by the SQUEEZE method^37^ as implemented in PLATON.^38^ Details of the structure determination and refinement of all compounds are summarized in Table S1 in the Supporting Information.

## 3 Results and Discussion

### 3.1 Mass Spectrometry

To evaluate the first stages of the clustering process, this work started with the analysis of the species present in solution upon the dissolution of ZrCl_4_ and the corresponding benzoic ligand (benzoic, 2-hydroxybenzoic, or 3-hydroxybenzoic acids) in anhydrous ethanol.^39−43^ The pH value of the resulting media was controlled by the addition of water to allow a precise control of the acidity. This variation exerts a strong influence on the species that are built up as confirmed by means of MS. In these studies, the appearances of pentameric and hexameric zirconium entities in which the carboxylic ligands are coordinated to the metal centers were detected. These species were later on isolated in the solid state and their crystal structures are also reported in this work.

**Figure 2:**
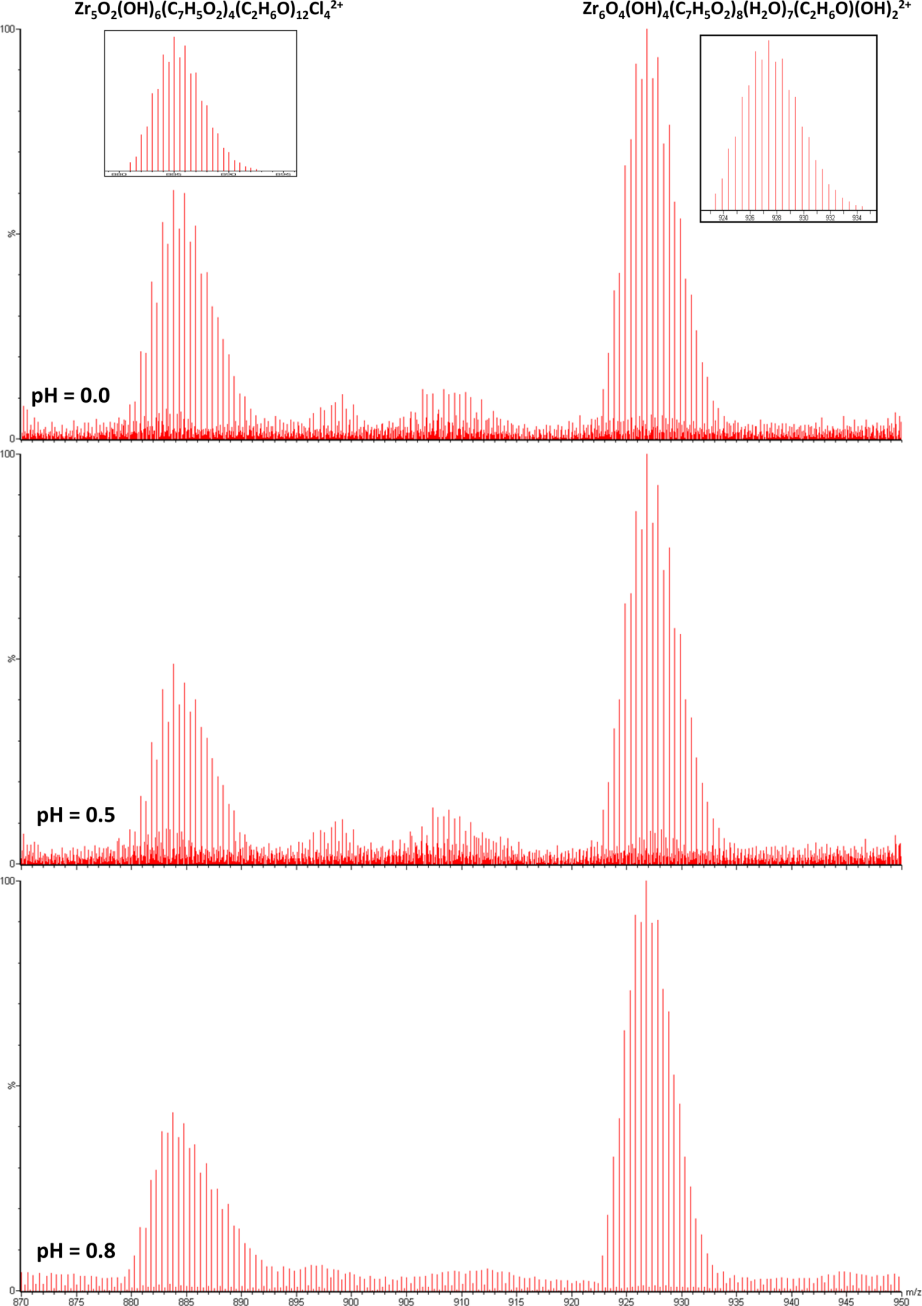
Influence of pH on the ESI^+^ MS spectra signals of the ZrCl_4_/benzoic acid system. Signals belonging to pentameric and hexameric zirconium species are identified in the *m*/*z*: 870–950 range.

Figure 2 shows the ESI^+^ mass spectra in the *m*/*z* 870–950 range obtained for the ZrCl_4_/benzoic acid system at different very acidic pH conditions (0, 0.5, and 0.8). All spectra show two major signals centered at *m*/*z* 884 and 927 with a 0.5 spacing of the peaks, indicative of 2+ charge states for both species. No species with charge greater than 2+ have been observed. The assigned molecular formula for the heavier signal is chemically sound and agrees well with the [Zr_6_(O)_4_(OH)_4_] core features of the ubiquitous zirconium-based hexameric SBU. However, the benzoate anions only partially occupy the peripheral positions around this core in such a way that the number of carboxylate groups attached to the cluster is reduced from the expected 12 (the well-known [Zr_6_(O)_4_(OH)_4_(OOC)_12_] SBU found in many MOFs) to 8 providing a carboxylate-unsaturated entity. This hexameric unsaturated species incorporates two additional hydroxide anions to provide the observed 2+ charge state, and solvent molecules complete the coordination sphere of the cluster.

**Scheme 1:**
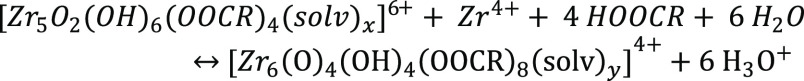
Acid–Base Equilibrium Governing the Transformation between Pentameric and Hexameric Species

Similarly, the signal centered at 884 has been assigned to a pentameric zirconium entity with 2+ charge in which 12 solvent molecules, 4 benzoato ligands, and 4 chlorides stabilize a [Zr_5_(O)_2_(OH)_6_] core. Interestingly, the signals assigned to the hexameric and pentameric species show important modifications in their relative intensities as a function of pH. The addition of water and subsequent dilution-driven mild basification of the media results in a relative decrease of the intensity for the signal belonging to the pentameric species, whereas that related to the hexameric species increases considerably. This fact can be interpreted as an acid–base equilibrium between the two polynuclear species according to the chemical reaction provided in Scheme 1.

The formula obtained for the pentameric species seems to be closely related to that of the hexameric cluster by releasing one zirconium atom and losing four benzoato ligands, and at the same time, reducing the amount of oxides and increasing the hydroxide amount to keep invariable the total amount of core bridging oxide/hydroxide ligands at eight. Thus, there is a probable connection between the zirconium oxide/hydroxide species that can be rationalized starting with the well-known [Zr_4_(OH)_8_(solv)_16_]^8+^ tetrameric entity,^44^ usually employed as a commercial reagent in the form of its chloride salt,^45^ which in the presence of carboxylic ligands evolves to a pentanuclear [Zr_5_(O)_2_(OH)_6_(OOC)_4_(solv)]^6+^ species. The greater polarization effect of the fifth zirconium(IV) center promotes the deprotonation of two of the hydroxides to provide a two oxide/six hydroxide core. Upon basification, these pentanuclear entities evolve into hexanuclear [Zr_6_(O)_4_(OH)_4_(OOC)_8_(H_2_O)]^4+^ species by the incorporation of a sixth zirconium that again increases the polarization at the hydroxide anions leading to a final four oxide/four hydroxide core. Further pH increases, favoring the deprotonation of the carboxylic ligands, would probably lead to the neutral [Zr_6_(O)_4_(OH)_4_(OOC)_12_] cluster found as an SBU in most of the zirconium-based MOFs.

The same studies performed using 2- and 3-hydroxybenzoic acid provided similar outcomes but with the presence of a greater dispersion of the pentameric and hexameric species due to variations on the solvent molecules (water and ethanol that interchange between them). They show the same 2+ *m*/*z* spacing in their signals and the described increase in the relative intensity of the hexameric species upon basification (see Section S5 of the Supporting Information). It is worth mentioning that the basification only implies a change from pH < 0 to pH ≈ 1.5.

Fortunately, these species were isolated in the solid state and a complete single-crystal XRD structural characterization was performed. As it will be shown below, the results fully corroborate the above described conclusion in such a way that we were able to isolate both pentameric [Zr_5_(O)_2_(OH)_6_(OOCR)_4_(H_2_O)_11_(HOR′)]Cl_6_ (R: C_6_H_5_; R′: C_2_H_5_, C_3_H_7_) and carboxylate-unsaturated hexameric [Zr_6_(O)_4_(OH)_4_(OOCR)_8_(H_2_O)_8_]Cl_4_ (R: C_6_H_5_ and C_6_H_5_O) compounds as a function of pH.

### 3.2 Crystal Structure of the Polynuclear Entities

**Figure 3:**
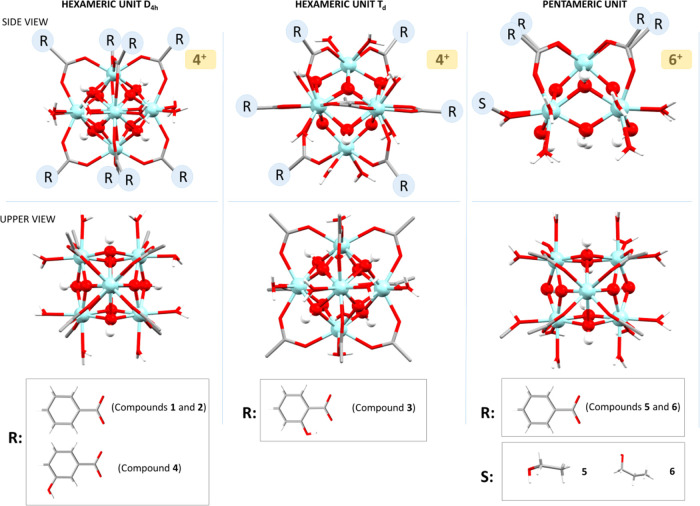
Summary of the polynuclear entities present in compounds **1**–**6**.

The crystal structure of compounds **1**–**6** contains the previously identified discrete polynuclear entities (hexameric for compounds **1**–**4**; pentameric for compounds **5** and **6**) in which the hydroxide or oxide anions are positioned alternately in the center of each triangular face of the metal defining the square pyramid or octahedron. It means the hexameric entity has a [Zr_6_(O)_4_(OH)_4_] core whereas the pentamer shows a [Zr_5_(O)_2_(OH)_6_] core in which the lack of a sixth zirconium atom implies that the four hydroxides pointing toward the vacant are not further polarized to produce the observed alternation of oxides/hydroxides of the triangular faces (Figure 3). The external coordination positions of the metal atoms, all of them showing a cubic antiprism geometry, are occupied by water molecules and carboxylate ligands. The hexameric and pentameric polynuclear entities have a 4+ and 6+ charge, respectively, balanced by chloride counterions. The coordination bond distances found follow the same trend for all the entities. Zr–O_oxide_ distances are always the shortest ones (2.00–2.14 Å), those involving the oxygen atoms of the carboxylate groups are between 2.16 and 2.28 Å, whereas those of hydroxides and water molecules are the longest ones with values between 2.18 and 2.37 Å. The distance between adjacent zirconium atoms within the polynuclear entities is in the range of 3.47–3.54 Å.

All compounds, except **5** and **6**, present a hexameric structure with the previously described octahedral geometry of the cluster in which instead of the expected 12 carboxylic ligands, only 8 are anchored to the hexanuclear Zr_6_(O)_4_(OH)_4_ core (benzoato in **1**, **2**; 2-hydroxybenzoato in **3**; and 3-hydroxybenzoato in **4**). The remaining coordination positions lacking carboxylate groups (herein after: carboxylate-unsaturated positions) are filled with water molecules. In spite of the apparent similarity between compounds **1**–**4**, the arrangement of the carboxylato ligands provides a source of isomerism in these octahedrally shaped polynuclear entities. In compounds **1**, **2** (benzoato), and **4** (3-hydroxybenzoato), the eight carboxylate groups bridge the equatorial and apical zirconium atoms. The coordination positions located at the equatorial edges of the octahedron located are occupied by water molecules affording a *D*_4*h*_ symmetry. The metal coordination environment at the apical position consists of two oxides, two hydroxides, and four oxygen atoms from carboxylate groups. However, the equatorial zirconium coordination environment consists of two oxides, two hydroxides, and two oxygen atoms from carboxylate groups and two water molecules.

In contrast, compound **3** (2-hydroxybenzoato ligand) shares many of the features of the previously described octahedral clusters, but the presence of the hydroxyl residue so close to the Zr_6_O_4_(OH)_4_ core implies that positioning four carboxylate groups around the apical zirconium positions is now disfavored and instead of the previously described *D*_4*h*_ arrangement, now a *T_d_* symmetry is achieved. In this new arrangement, all the equatorial edges are occupied by the carboxylato ligands but only half of the equatorial–apical linking edges are occupied in an alternated way. Alternatively, it can be described focusing on the carboxylate lacking edges that are arranged in a tetrahedral disposition. Therefore, the coordination environment of the apical zirconium is composed of two oxide molecules, two hydroxides, two water molecules, and two carboxylate groups. On the other hand, the equatorial zirconium coordination environment consists of two oxides, two hydroxides, three carboxylate groups, and a single water molecule. As usually happens for 2-hydroxybenzoate anions and 2-hydroxybenzoic acid molecules, an intramolecular hydrogen bond between the hydroxyl residue and one of the carboxylate/carboxylic oxygen atoms is observed.

Compounds **5** and **6** (with benzoato ligands) consist of square-based pyramidal pentameric entities in which the absence of the sixth zirconium atom is accompanied with the reduction of the number of anchored carboxylato ligands from eight to four. These four carboxylato ligands are located bridging the zirconium atoms in the basal plane with the apical one. The lack of the sixth zirconium atom also exerts its influence in a lower polarization capacity and instead of the four oxide/four hydroxide composition for the polynuclear core, a two oxide/six hydroxide ratio is observed. Again, the remaining positions to complete the eight coordination environment of the zirconium atoms are occupied by water molecules. As a result, the coordination environment of the apical zirconium is composed of two oxides, two hydroxides, and four oxygen atoms from four carboxylate groups, whereas the coordination environment consists of one oxide, three hydroxides, a single carboxylate oxygen atom, and three water molecules for three of the four basal plane zirconium atoms. The fourth zirconium in the basal plane shows a different coordination and gets coordinated to an alcohol molecule (ethanol in compound **5** and propanol in compound **6**). Therefore, instead of having three coordinated water molecules, it only presents two and the third one is replaced by the alcohol molecule.

**Figure 4:**
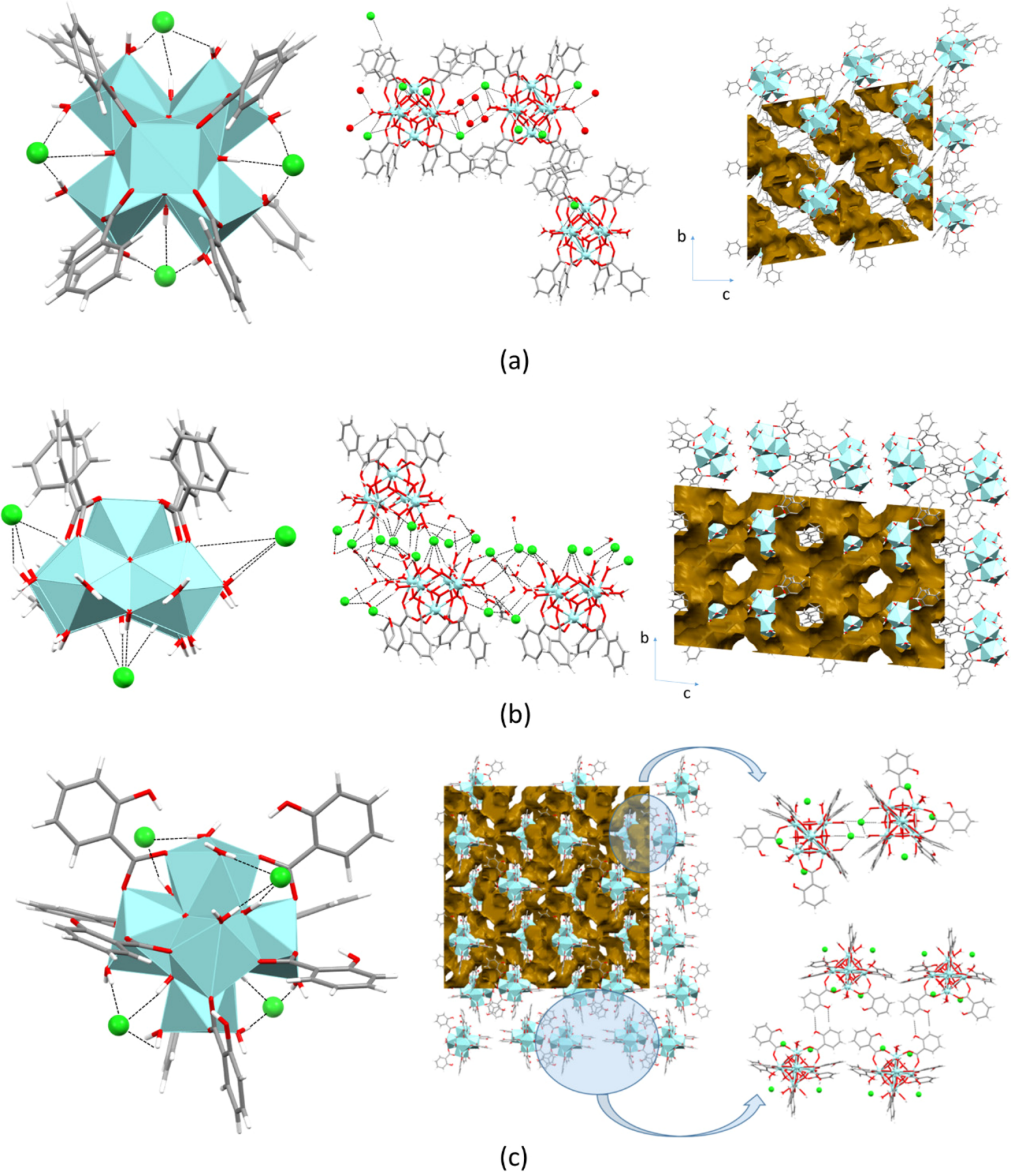
Structural features of compounds **1** (a), **5** (b), and **3** (c). The brownish surfaces depicted in the packing images represent the volume occupied by the noncoordinated solvent molecules.

The cationic nature of the polynuclear entities implies that the ionic interactions with the chloride counterions play a key role in directing the crystal packing of these compounds. In compounds **1**–**3**, ionic interactions are also reinforced by strong hydrogen bonds established by chloride counterions and the bridging hydroxide anions located in half of the triangular faces of the octahedrally shaped hexameric entities (*d*_OH···Cl_: 2.97–3.26 Å). These chloride counterions are tetrahedrally arranged around the metal–organic clusters but the size difference between the big cationic entities and the comparatively small chloride anions make it difficult to achieve a strong packing of the crystal structure and thus requires a huge amount of solvent molecules to provide some cohesiveness to the overall 3D architecture (Figure 4). In the case of compound **4** (3-hydroxybenzoato), the hydroxyl residues protruding from the hexameric entities provide a better placement for the chloride counterions to establish hydrogen bonding interactions. These features also provide an interaction pathway between the neighboring hexameric entities, which involves 3-hydroxybenzoato ligands and the coordinated water molecules as hydrogen-bond donors toward the chloride counterions. Unfortunately, these interactions only spread along the (101) crystallographic plane and the 3D cohesiveness requires again a great amount of solvent molecules.

The 6+ charge of the pentameric species implies the presence of six chloride counterions but only three of them are strongly hydrogen bonded to the bridging hydroxide anions. Two of them, as previously described for most of the hexameric entities, imply the interaction with the hydroxides placed in the triangular faces and the third one occupies a position close to what would be the sixth zirconium atom if the cluster would have evolved toward the hexameric entity. In this position, the chloride anion acts as an acceptor of the hydrogen bonding interactions involving the four bridging hydroxides located in the basal plane of the cluster. The remaining three chloride counterions are more loosely interacting with the cluster through the coordinated water molecules. In fact, one of these chlorides is disordered over two positions indicative of the less specific interactions they establish.

In all compounds, solvent molecules account for a great portion of the total volume of the solid (21.9–33.3%) and their loss after the removal of the crystals from the mother liquid implies a transformation of the crystal structure. In fact, these great amounts of solvent molecules seem to saturate the capacity of the aromatic monocarboxylic ligands to establish supramolecular interactions, and no π-stacking has been observed among them. Only compound **3** exhibits some weak double C–H···O hydrogen bonds involving the 2-hydroxibenzoato ligands from adjacent hexanuclear entities. The fluidity of the supramolecular network involving such a huge amount of solvent molecules allows us to isolate compounds **1** and **2**, which can be considered two solvation stages of the same compound, in spite of the fact that they exhibit completely different unit cell parameters and space group. All in all, although the structure collapse makes the characterization of bulk samples difficult, the homogeneity of the crystalline phase was assessed by PXRD analyses over samples introduced in Lindemann tubes, and the stability of the clusters upon their removal from mother liquors was addressed on the basis of solid state ^1^H- and ^13^C-MAS-NMR spectroscopy (see the details in the Supporting Information).

## 4 Conclusions

In summary, we have shown that there is plenty of chemistry still to be discovered about the first stages of the zirconium-carboxylate cluster formation. The results rendered here must be understood as frozen images along this process but also reveal the opportunities that arise from a fine control of the synthetic conditions. In fact, among the intermediate species that can be found in the formation of Zr-carboxylate clusters, a structure in which the 12 carboxylato ligands bridge the 12 Zr–Zr edges is the lowest in energy according to quantum mechanical calculations.^46^ However, this report does not take into account the specific synthetic conditions at which these entities grow. Our work confirms that small modifications of the pH of the media can considerably affect in the isolation of clusters with different nuclearities and carboxylate-ligand contents. In addition, species with unsaturated carboxylate and/or metal positions can be a starting point to develop a richer chemistry by completing these vacancies with different carboxylic ligands or metal centers.^47^ In this sense, it has been possible to isolate zirconium hexameric entities with only eight carboxylate groups attached to the cluster and the remaining free four carboxylate positions arranged in a square planar or tetrahedral disposition. An unprecedented pentameric entity closely related to the hexameric ones by the release of one of the apical zirconium positions is also achieved, being able to reveal the acid–base nature and pH dependence of the transformation between both species.

As a final remark, it is worth to mention that the results obtained here reveal that in spite of the cationic nature of the zirconium clusters reported herein, their chemistry shows some resemblance with that of the anionic polyoxometalates (POMs), as the acidity of the media is also here a key factor in the final nuclearities of the resulting clusters.^48^ However, differences arise from the fact that the oxidation state of the zirconium cation is not as high as those displayed by the typical addenda metals in POMs (mainly V, Mo, and W). This implies that the polarizing capability of zirconium is not that high and it cannot promote the complete deprotonation of all the coordinated water molecules to afford an oxide rich environment able to stabilize these metal ions. Instead of this, the formation of zirconium-based entities with higher nuclearities than the classical [Zr_4_(OH)_8_(H_2_O)_16_]^8+^ cation will require the presence of coordinated carboxylate groups bridging and holding together the metal centers.

## Supporting Information Available

Details of spectroscopic data (FTIR, solid state ^1^H-NMR and ^13^C-NMR, and mass spectroscopy), thermogravimetric measurements, PXRD data collection, and crystallographic and structural data (PDF)
