# Supplementary material for: The Chemistry of Zirconium/Carboxylate Clustering Process: Acidic Conditions to Promote Carboxylate-Unsaturated Octahedral Hexamers and Pentanuclear Species
Source: Inorg Chem. 2022 Mar 14;61(12):4842–51. doi: 10.1021/acs.inorgchem.1c03466 (PMC9993394; doi:10.1021/acs.inorgchem.1c03466)
Supplement: Supplementary file 1 — ic1c03466_si_001.pdf [file ic1c03466_si_001.pdf]

Supporting Information for

# The chemistry of zirconium/carboxylate clustering process: acidic conditions to promote carboxylate-unsaturated octahedral hexamers and pentanuclear species

*Jon Pascual-Colino,*[a] *Beñat Artetxe,*[a] *Garikoitz Beobide,*[a,b] *Oscar Castillo,*[a,b,*] *Maria Luz Fidalgo-Mayo,*[c] *Ainhoa Isla-López,*[a] *Antonio Luque,*[a,b] *Sandra Mena-Gutiérrez,*[a] *Sonia Pérez-Yáñez*[b,c]

Corresponding autor: oscar.castillo@ehu.eus

[a]Departamento de Química Orgánica e Inorgánica, Facultad de Ciencia y Tecnología, Universidad del País Vasco/Euskal Herriko Unibertsitatea, UPV/EHU, Apartado 644, E-48080 Bilbao, Spain.

[b]BCMaterials, Basque Center for Materials, Applications and Nanostructures, UPV/EHU Science Park, E-48940 Leioa, Spain.

[c]Departamento de Química Orgánica e Inorgánica, Facultad de Farmacia, Universidad del País Vasco/Euskal Herriko Unibertsitatea, UPV/EHU, E-01006 Vitoria-Gasteiz, Spain.

S1. POWDER X-RAY DIFFRACTION .......... 3
S2. CRYSTAL STRUCTURE OF COMPOUNDS **1–6** .......... 10
S3. FOURIER TRANSFORM INFRARED SPECTRA (FTIR) .......... 13
S4. THERMOGRAVIMETRIC ANALYSIS .......... 18
S5. MASS SPECTROMETRY (MS) .......... 24
S6. SOLID STATE NUCLEAR MAGNETIC RESONANCE (NMR) .......... 38
S7. REFERENCES .......... 45

S1. POWDER X-RAY DIFFRACTION

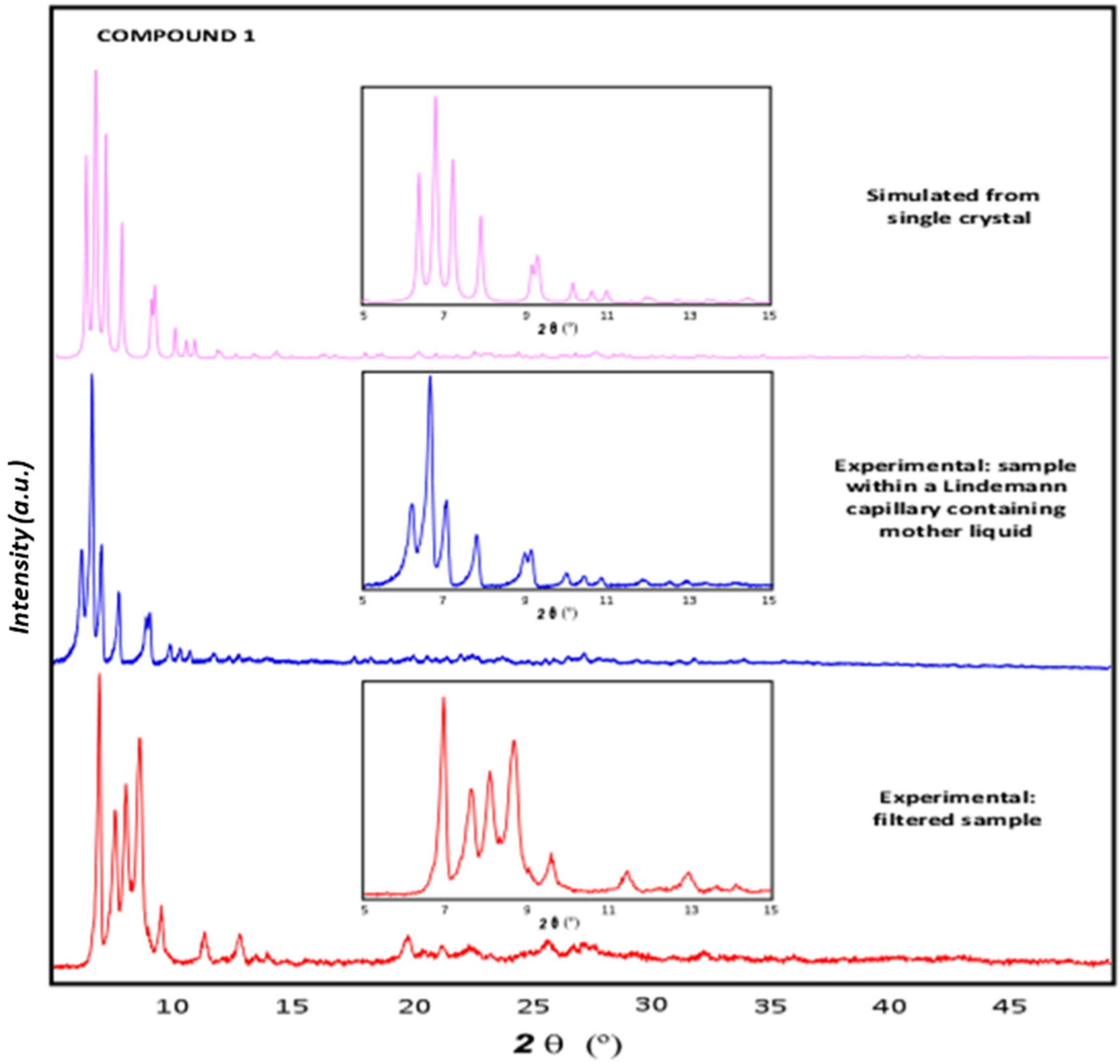


**Figure S1.** Powder X-ray diffraction patterns: simulated pattern from single crystal of compound **1** (top), experimental pattern measured within the mother liquid using a Lindemann capillary (middle) and experimental pattern measured on filtered sample (bottom). Depicted in the insets an amplification of the 5–15° area for a better comparison.

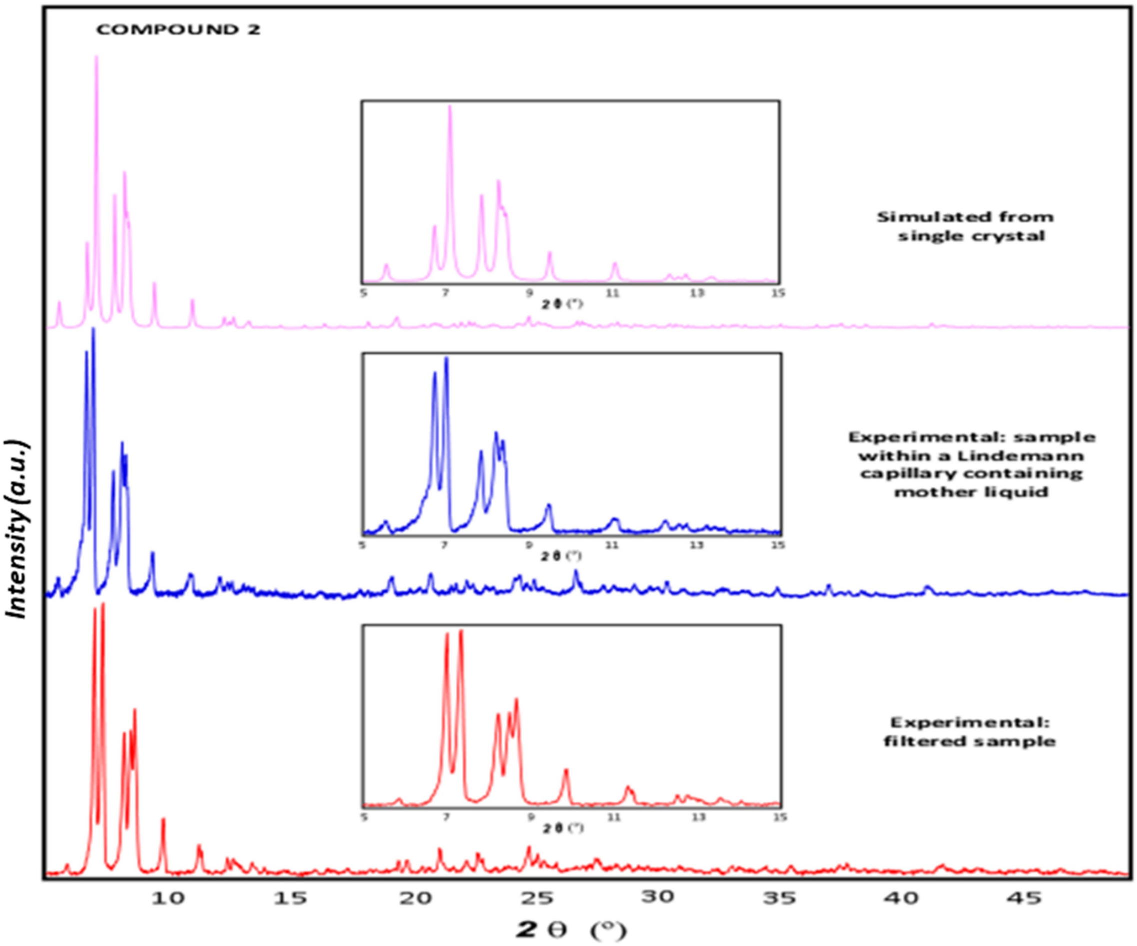


**Figure S2.** Powder X-ray diffraction patterns: simulated pattern from single crystal of compound **2** (top), experimental pattern measured within the mother liquid using a Lindemann capillary (middle) and experimental pattern measured on filtered sample (bottom). Depicted in the insets an amplification of the 5–15° area for a better comparison.

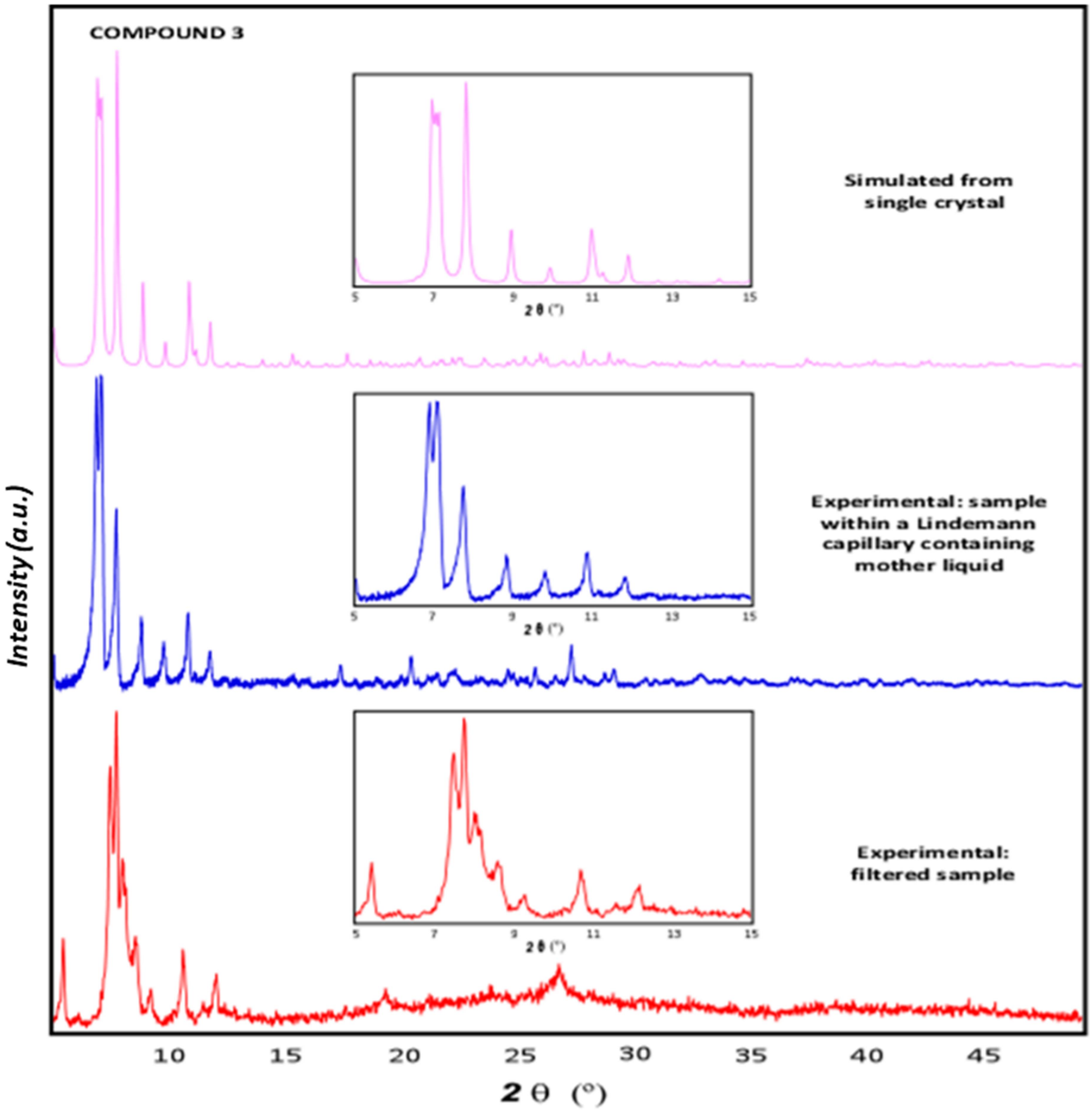


**Figure S3.** Powder X-ray diffraction patterns: simulated pattern from single crystal of compound **3** (top), experimental pattern measured within the mother liquid using a Lindemann capillary (middle) and experimental pattern measured on filtered sample (bottom). Depicted in the insets an amplification of the 5–15° area for a better comparison.

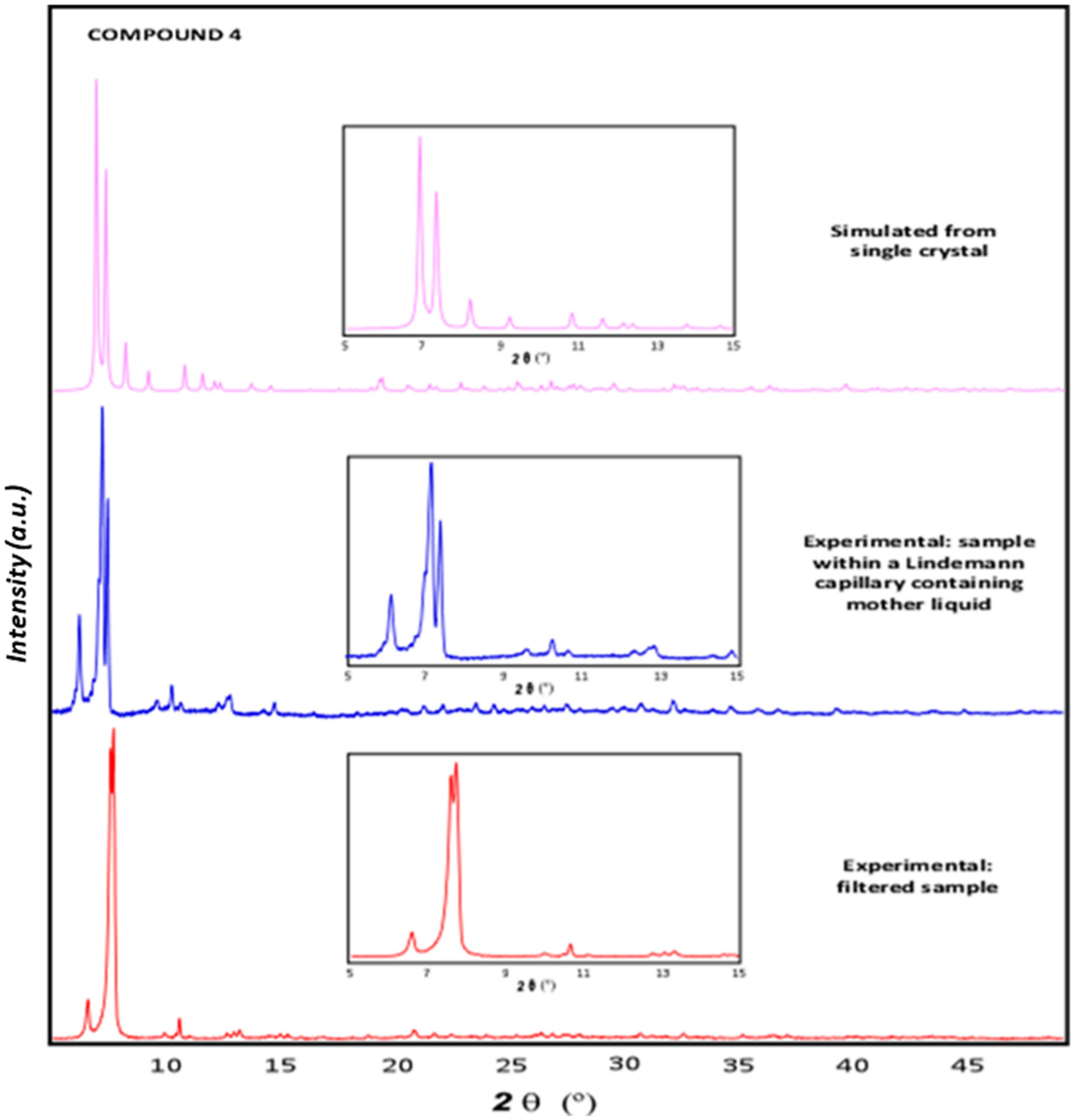


**Figure S4.** Powder X-ray diffraction patterns: simulated pattern from single crystal of compound **4** (top), experimental pattern measured within the mother liquid using a Lindemann capillary (middle) and experimental pattern measured on filtered sample (bottom). Depicted in the insets an amplification of the 5–15° area for a better comparison.

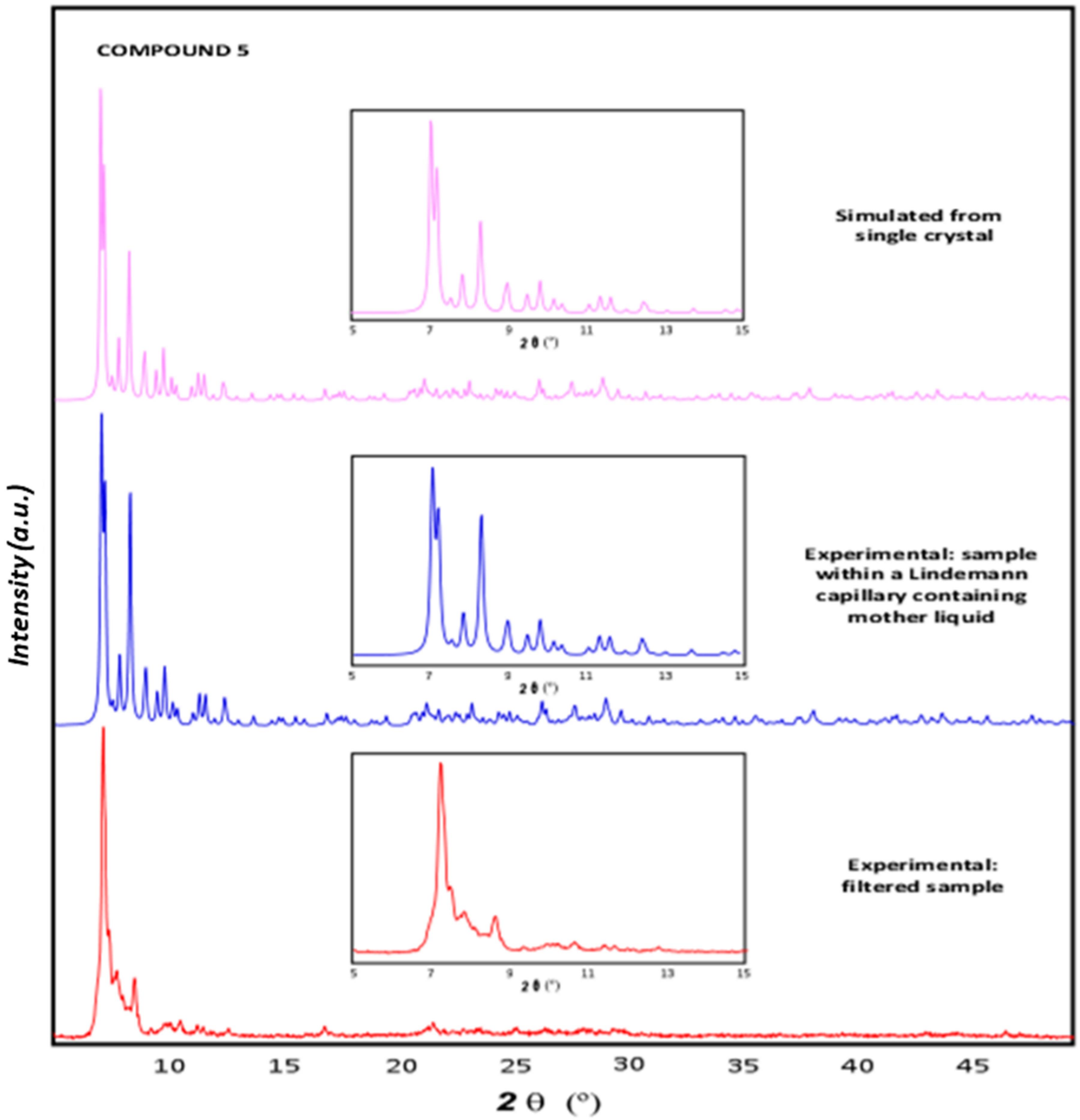


**Figure S5.** Powder X-ray diffraction patterns: simulated pattern from single crystal of compound **5** (top), experimental pattern measured within the mother liquid using a Lindemann capillary (middle) and experimental pattern measured on filtered sample (bottom). Depicted in the insets an amplification of the 5–15° area for a better comparison.

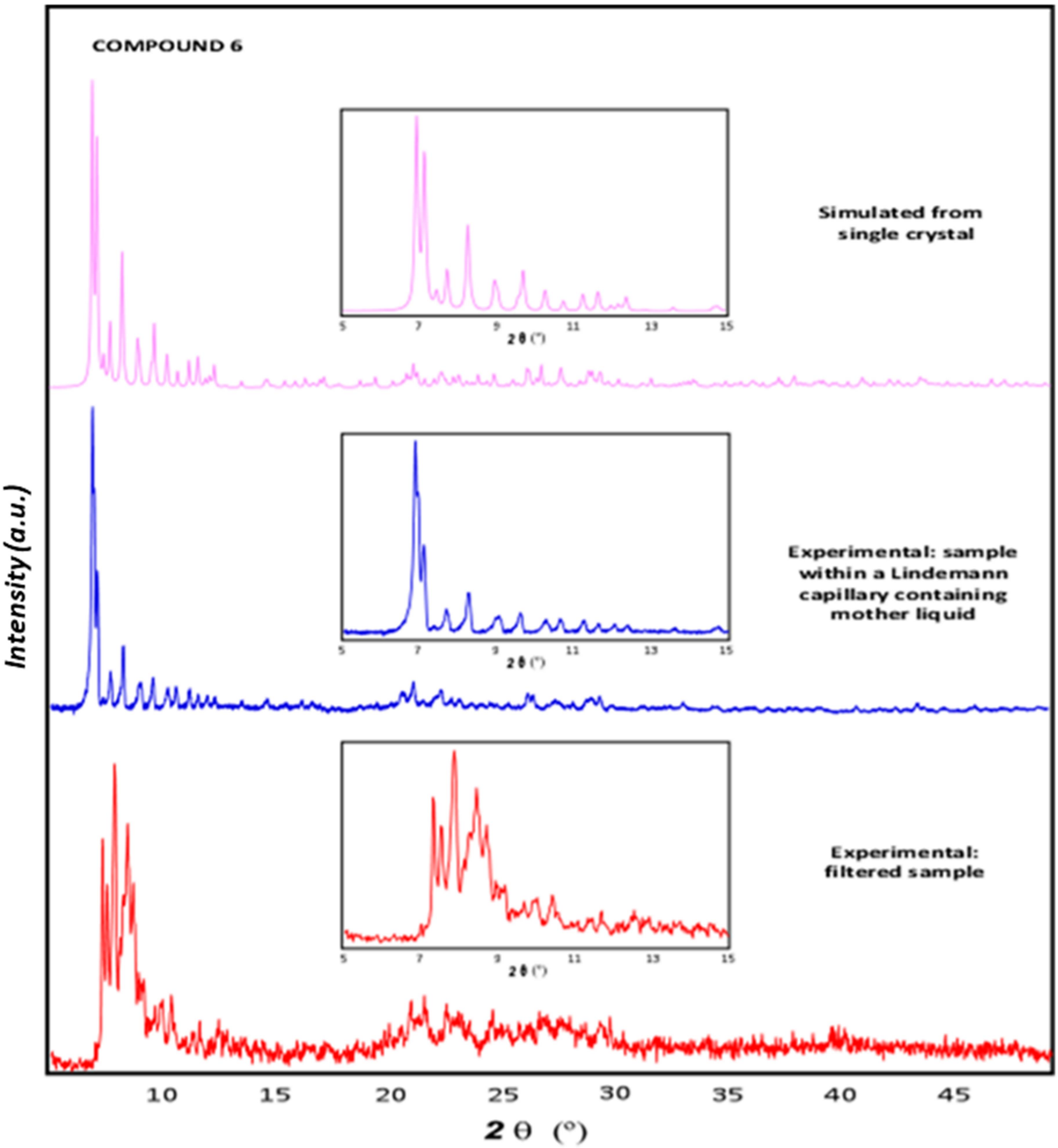


**Figure S6.** Powder X-ray diffraction patterns: simulated pattern from single crystal of compound **6** (top), experimental pattern measured within the mother liquid using a Lindemann capillary (middle) and experimental pattern measured on filtered sample (bottom). Depicted in the insets an amplification of the 5–15° area for a better comparison.

## S2. CRYSTAL STRUCTURE OF COMPOUNDS **1**–**6**

**Table S1.** Crystallographic data and structure refinement details of compounds **1**−**6**.[a,b]

| | **1** | **2** | **3** | **4** | **5** | **6** |
|---|---|---|---|---|---|---|
| empirical formula | $C_{58}H_{136}Cl_4O_{68}Zr_6$ | $C_{56}H_{90}Cl_4O_{47}Zr_6$ | $C_{56}H_{116}Cl_4O_{68}Zr_6$ | $C_{56}H_{114}Cl_4O_{67}Zr_6$ | $C_{34}H_{85}O_{40}Cl_6Zr_5$ | $C_{37}H_{93}Cl_6O_{41}Zr_5$ |
| formula weight | 2610.78 | 2204.39 | 2566.60 | 2548.59 | 1802.81 | 1862.91 |
| crystal system | Monoclinic | Triclinic | Monoclinic | Monoclinic | Triclinic | Triclinic |
| space group | *Cc* | $P\bar{1}$ | $P2_1/n$ | $I2/m$ | $P\bar{1}$ | $P\bar{1}$ |
| *a* | 27.4376(7) | 14.5136(11) | 15.7858(4) | 13.8590(2) | 12.8221(6) | 12.8348(3) |
| *b* | 27.6947(8) | 16.1144(8) | 25.2488(7) | 24.8654(3) | 12.8803(5) | 12.9736(3) |
| c | 27.7040(9) | 19.1548(11) | 25.1063(7) | 15.1787(2) | 21.7800(8) | 21.9129(4) |
| α | 90 | 80.923(4) | 90 | 90 | 92.867(3) | 93.420(2) |
| β | 108.540(3) | 79.585(5) | 97.311(3) | 98.1800(10) | 100.776(4) | 101.463(2) |
| γ | 90 | 89.049(5) | 90 | 90 | 101.501(4) | 98.994(2) |
| V (Å$^3$) | 19959.1(11) | 4350.6(5) | 9925.3(5) | 5177.51(12) | 3448.4 | 3516.50(14) |
| Z | 8 | 2 | 4 | 2 | 2 | 2 |
| T (K) | 100.01(10) | 150.00(10) | 100.00(10) | 150.00(2) | 150.00(10) | 150.00(10) |
| λ (Å) | 0.71073 | 0.71073 | 0.71073 | 1.54184 | 0.71073 | 1.54184 |
| $\rho_c$ (g·cm$^{-3}$) | 1.738 | 1.683 | 1.718 | 1.635 | 1.736 | 1.759 |
| $\mu$ (cm$^{-1}$) | 0.821 | 0.908 | 0.824 | 6.653 | 1.053 | 8.804 |
| S$^a$ | 0.792 | 1.008 | 1.009 | 1.084 | 0.880 | 1.034 |
| $R_{int}$ | 0.0890 | 0.0700 | 0.1311 | 0.0399 | 0.0664 | 0.0301 |
| **Final R indices** | | | | | | |
| [I > 2σ(I)] $R_1^b/wR_2^c$ | 0.0751/0.1948 | 0.0842/0.1975 | 0.0673/0.1519 | 0.0602/0.1828 | 0.0608/0.1371 | 0.0505/0.1426 |
| all data $R_1^b/wR_2^c$ | 0.1896/0.2267 | 0.1762/0.2672 | 0.1501/0.1976 | 0.0686/0.1925 | 0.1278/0.1527 | 0.0544/0.1462 |

$^a$S=$[\sum w(F_0^2-F_c^2)^2/(N_{obs}-N_{param})]^{1/2}$. $^b R_1=\sum\|F_0|-|F_c\|/\sum|F_0|$. $^c wR_2 = [\sum w(F_0^2-F_c^2)^2/\sum wF_0^2]^{1/2}$; $w = 1/[\sigma^2(F_0^2) + (aP)^2 + b]$ where $P = (\max(F_0^2,0) + 2F_c^2)/3$; **1** (a = 0.1171, b =0); **2** (a = 0.0973, b =0); **3** (a = 0.0642, b = 0); **4** (a = 0.1109, b = 38.7354); **5** (a = 0.0575, b = 0); **6** (a = 0.0793, b =21.0678).

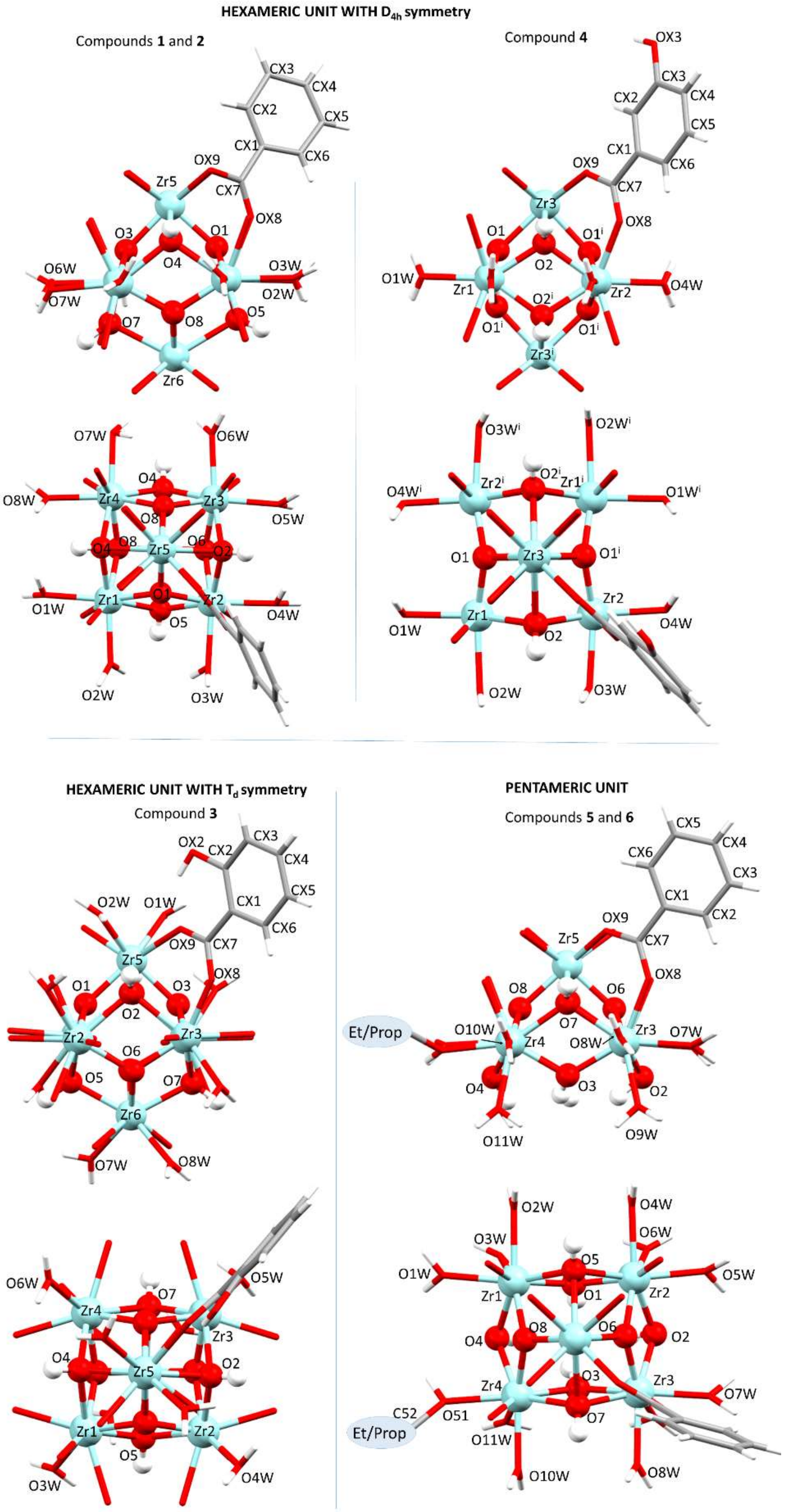


**Figure S7.** Zirconium cluster of compounds **1**–**6** showing the numbering scheme. For the sake of clarity, only one of the coordinated carboxylato ligands is represented.

S3. FOURIER TRANSFORM INFRARED SPECTRA (FTIR)

The infrared spectra of the reported Zr-carboxylato compounds and the assignation of the vibration modes are gathered in Figure S8 and Table S2.

The analysis performed over dry samples shows, in all cases, the presence of the aromatic carboxylic ligands and the signals arising from the cluster. At high frequencies, in the 3600–2800 $cm^{-1}$ spectral region, all the spectra exhibit a broad and intense band centered at ~3380 $cm^{-1}$ that corresponds to the stretching of O–H bond from water molecules. Intense vibrational bands around 1600 $cm^{-1}$ are attributed to the antisymmetric stretching vibrations of the carboxylate groups whereas the symmetric stretching vibrations of the carboxylate groups appear around 1400 $cm^{-1}$. The presence of the hydroxyl group in compounds **3** and **4** is confirmed by the presence of an additional band at 1231–1253 $cm^{-1}$ corresponding to its in-plane vibration mode. The remaining bands at lower frequencies are attributed to the bending modes of the aromatic rings and the stretching of $Zr–O_{oxide/hydroxide/carboxylate}$ bonds.

**Table S2.** FTIR assignation of compounds **1-6**.[1-4]

| Benzoic acid | 2/3-Hydroxybenzoic | Comp. 1 | Comp. 2 | Comp. 3 | Comp. 4 | Comp. 5 | Comp. 6 | Assignation[a,b] |
|---|---|---|---|---|---|---|---|---|
| 3400s | 3400s | 3370vs | 3410vs | 3340s | 3380s | 3420vss | 3410vs | υ O—H, υ C—H |
| | | | | | | 1622w/1640w | 1622w/1640w | |
| 1700s | 1662s | 1600s | 1600s | 1622s | 1608s | 1600s | 1600s | $υ_{as}$ COO |
| 1580s | 1558s/1610s | 1555s/1525s | 1555s/1528vs | 1586s/1551s | 1564s/1533w | 1560m/1531vs | 1560s/1520vs | υ C=C |
| 1495s | 1501s/1450s | 1495s | 1490s | 1484m/1466s | 1493w/1448s | 1490m | 1490s | υ C—C |
| 1432f | 1387s | 1420vs | 1420vs | 1395vs | 1413s | 1415vs | 1410vs | $υ_s$ COO |
| 1298F | 1315m | 1305m | 1300m | 1311s | 1302s | 1310m | 1306m | υ C—O |
| | 1200s | | | 1244vs | 1253s/1231w | | | $δ_{ip}$ O—H |
| 1158m/1178m | 1159s | 1180s/1155m | 1180s/1155m | 1160s/1144s | 1160m/1120s | 1175m/1152w | 1180m/1155w | $δ_{ip}$ C—$H_{arom}$ |
| 1056m | 1085m | 1065s | 1070s | 1097m | 1075s | 1070m | 1070m | $δ_{ip}$ CCH |
| 1014m | 1014m | 1025s | 1020s | 1026s | 1000s | 1018m | 1020m | $δ_{ring}$ |
| 948s | 948s | 935m | 930m | 951m | 942s | 942m | 940m | $δ_{oop}$ O—H |
| 830m | 808s | 840w | 840w | 808s | 795s | 840w | 840w | υ C—C |
| 724s | 759s | 720s | 720s | 755vs | 764vs | 720s | 720vs | $δ_{ip}$ C—H, $υ_{as}$ Zr—O—Zr |
| 655f | | 660vs | 653vs | 648m | 657vs | 674s/650m | 671s/650m | υ Zr—$O_{carbo}$, $δ_{ip}$ C—H |
| | | | | | | 524w | 519w | υ Zr—$O_{carbo}$, Zr—O |
| | | 465m | 460m | 475w/422w | 457m | 458m | 470m | Zr—O |

[a]vs: very strong, s: strong, m: medium, w: weak. [b]s: symmetric, as: antisymmetric, υ: stretching vibration, δ: bending vibration, ip = in plane, oop = out of plane.

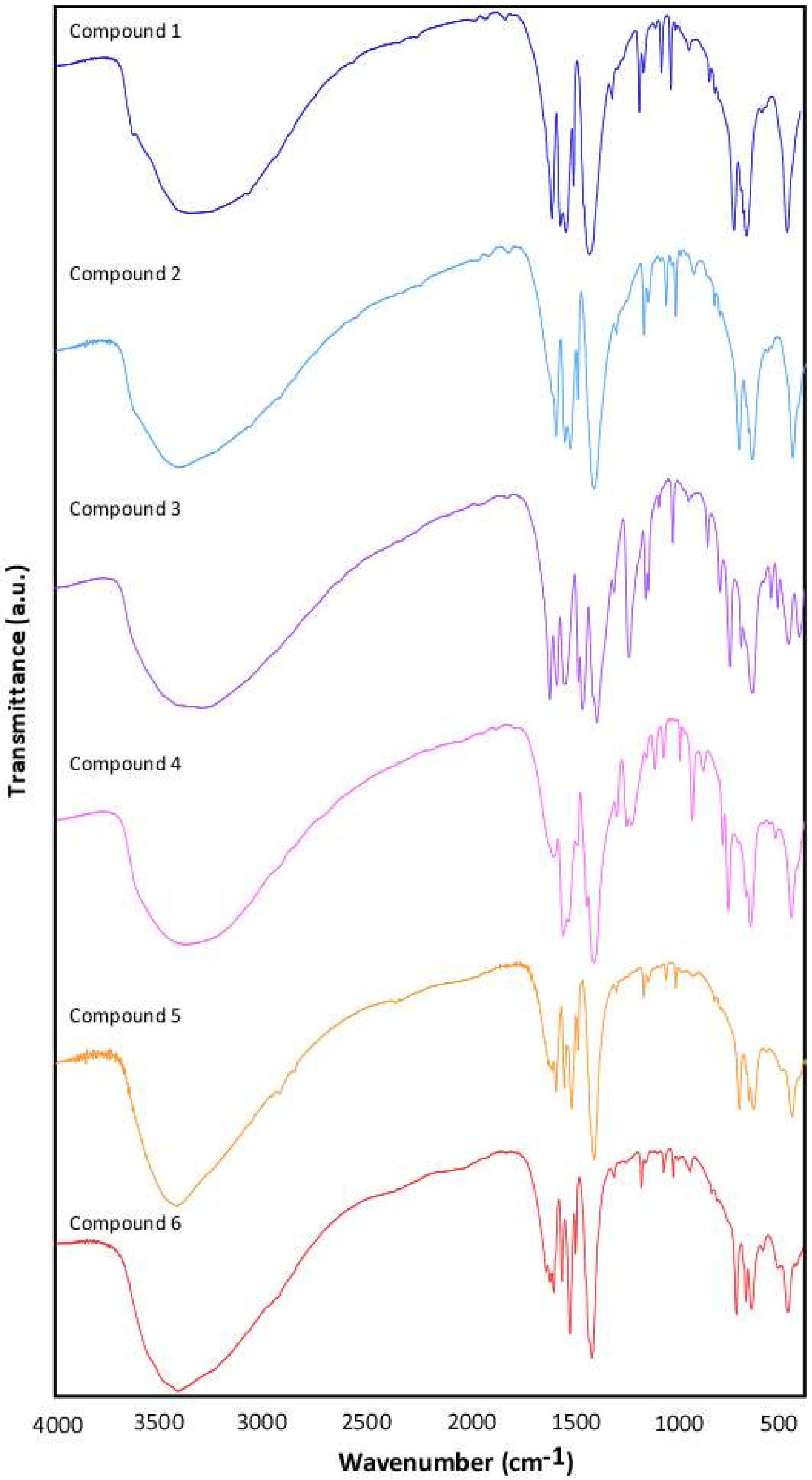


**Figure S8.** FTIR spectra of compounds **1**–**6**.

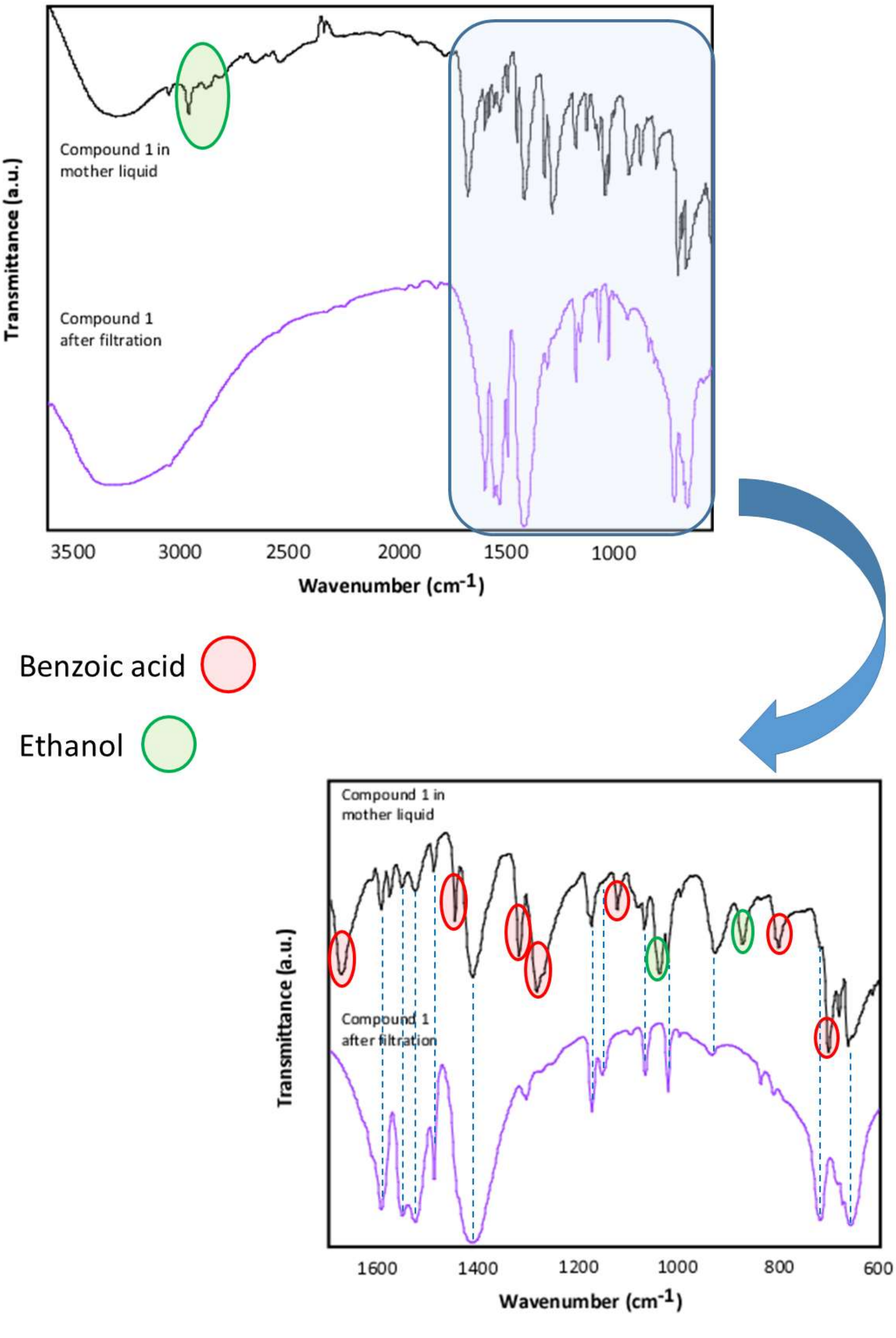


**Figure S9.** FTIR spectra of compounds **1** in mother liquid (black line) and the comparison with the spectra done after filtration (purple line). Bands assigned to benzoic acid and ethanol present in the mother liquour are colored in red and green, respectively.

## S4. THERMOGRAVIMETRIC ANALYSIS

Thermogravimetric analysis of compounds (**1**–**6**) were performed in synthetic air from 30 °C to 800 °C with a temperature increase rate of 5 °C/min (Figures S10–S12; Table S3). In all compounds, the thermograms show three main weight loss stages. First, crystallization solvent molecules are released at a temperature range of 25 – 85 °C. Thereafter, the compounds lose the coordination water molecules at 85 – 250 °C range and the release the chloride anions as hydrogen chloride takes place by acquiring a proton from the four hydroxide groups of the corresponding cluster. After that, the framework decomposition takes place to lead to $ZrO_2$ as final residue at 500 °C. It corresponds to a mixture of the monoclinic (PDF reference code 01-072-0597) and tetragonal (PDF reference code 01-071-1282) phases of $ZrO_2$.

**Table S3.** Thermoanalytic data for compounds **1-6**.[a]

| Step | $T_i$ | $T_{peak}$ | $T_f$ | Δm(%) | ΣΔm(%) | $ΣΔm(\%)_{theor}$ |
|---|---|---|---|---|---|---|
| **Comp. 1** | | | | | | |
| **1** | 25 | 50 | 70 | 9.00 | 9.00 | 9.15 (-10 $H_2O$) |
| **2** | 95 | | 115 | 3.60 | 12.60 | 12.86 (-2 HCl) |
| **3** | 115 | 140 | 145 | 4.10 | 16.70 | 16.56 (-2 HCl) |
| **4** | 330 | 505 | 800 | 45.78 | 62.48 | 62.59 ($ZrO_2$) |
| **Comp. 2** | | | | | | |
| **1** | 25 | 60 | 85 | 9.10 | 9.10 | 9.21 (-11 $H_2O$) |
| **2** | 85 | 140 | 160 | 10.45 | 19.55 | 19.45 (–8 $H_2O$ + –2HCl) |
| **3** | 160 | | 250 | 3.18 | 22.73 | 22.83(–2HCl) |
| **4** | 250 | 410 | 800 | 42.57 | 65.3 | 65.23 ($ZrO_2$) |
| **Comp. 3** | | | | | | |
| **1** | 25 | 90 | 125 | 17.78 | 17.78 | 17.94 (-25 $H_2O$) |
| **2** | 125 | 150 | 180 | 11.45 | 29.23 | 29.48 (–8 $H_2O$ + –4HCl) |
| **3** | 180 | 396 | 800 | 41.35 | 70.58 | 70.62 ($ZrO_2$) |
| **Comp. 4** | | | | | | |
| **1** | 25 | 55 | 85 | 15.69 | 15.69 | 15.46 (-21 $H_2O$) |
| **2** | 85 | 165 | 250 | 11.81 | 27.5 | 27.88 (–8 $H_2O$ + –4HCl) |
| **3** | 350 | 490 | 800 | 42.27 | 69.77 | 69.78 ($ZrO_2$) |
| **Comp. 5** | | | | | | |
| **1** | 25 | 35 | 75 | 8.87 | 8.87 | 9.14 (-6 $H_2O$ + ethanol) |
| **2** | 75 | 80 | 95 | 4.38 | 13.25 | 13.47 (2 HCl) |
| **3** | 95 | 120 | 165 | 14.64 | 27.89 | 28.02 (11 $H_2O$ + ethanol) |
| **4** | 165 | 508 | 800 | 35.56 | 63.45 | 63.42 ($ZrO_2$) |
| **Comp. 6** | | | | | | |
| **1** | 25 | 60 | 65 | 9.10 | 9.10 | 9.42 (–9 $H_2O$) |
| **2** | 110 | 100 | 115 | 16.13 | 25.23 | 25.32 (–11 $H_2O$ + –2HCl) |
| **3** | 150 | 495 | 800 | 38.63 | 63.86 | 63.87 ($ZrO_2$) |

[a] $T_i$ = initial temperature; $T_p$ = DTA peak temperature; $T_f$ = final temperature; Δm(%) = mass loss percentage for each process; ΣΔm(%) = total mass loss percentage; $ΣΔm(\%)_{theor}$ = theoretical total mass loss percentage.

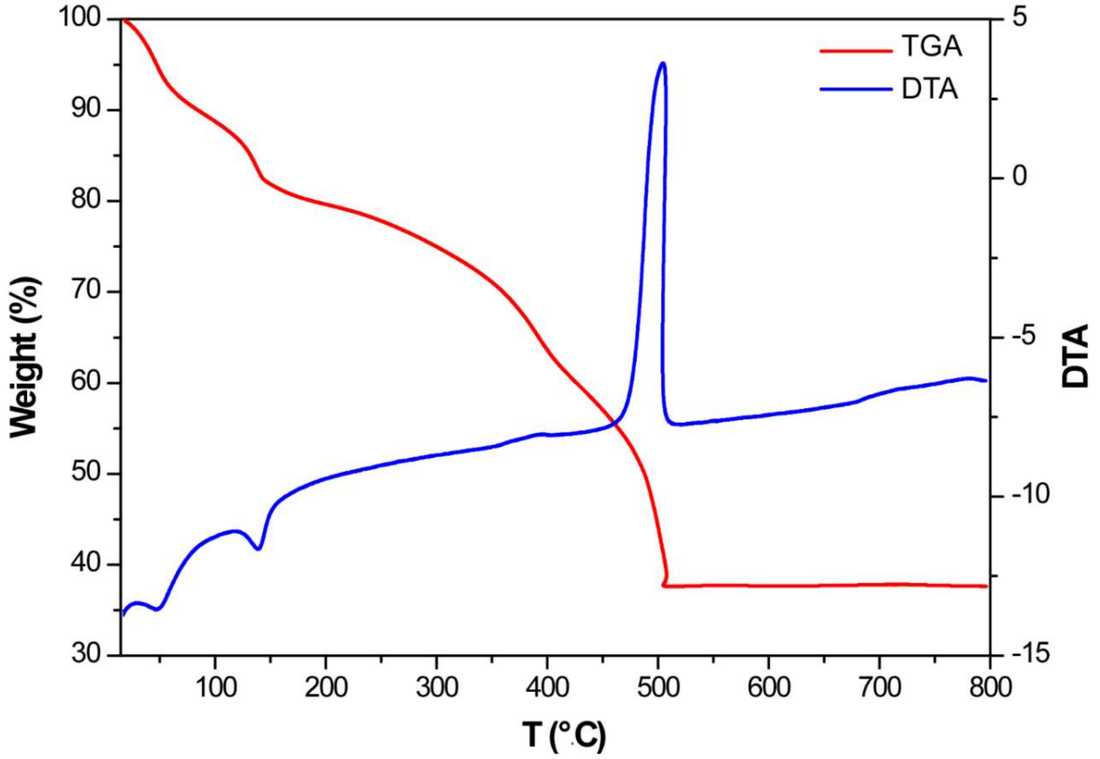


(a)

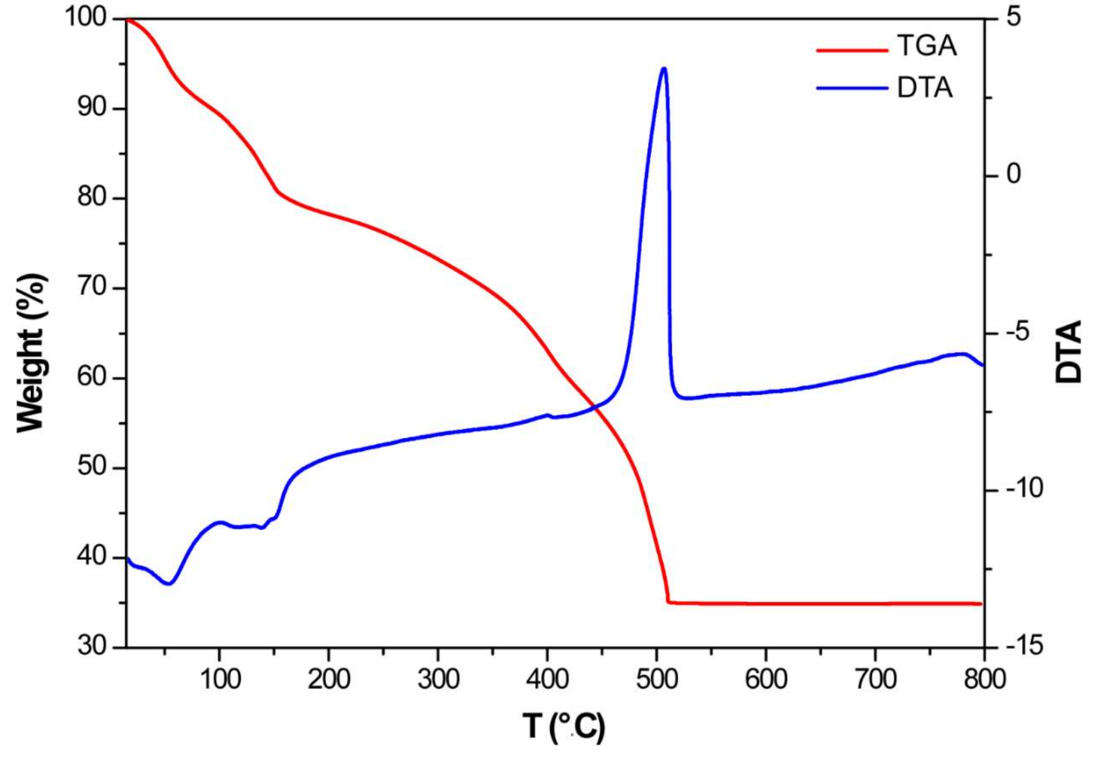


(b)

**Figure S10.** Thermogravimetric data (TGA-DTA curves) for compounds **1** (a) and **2** (b).

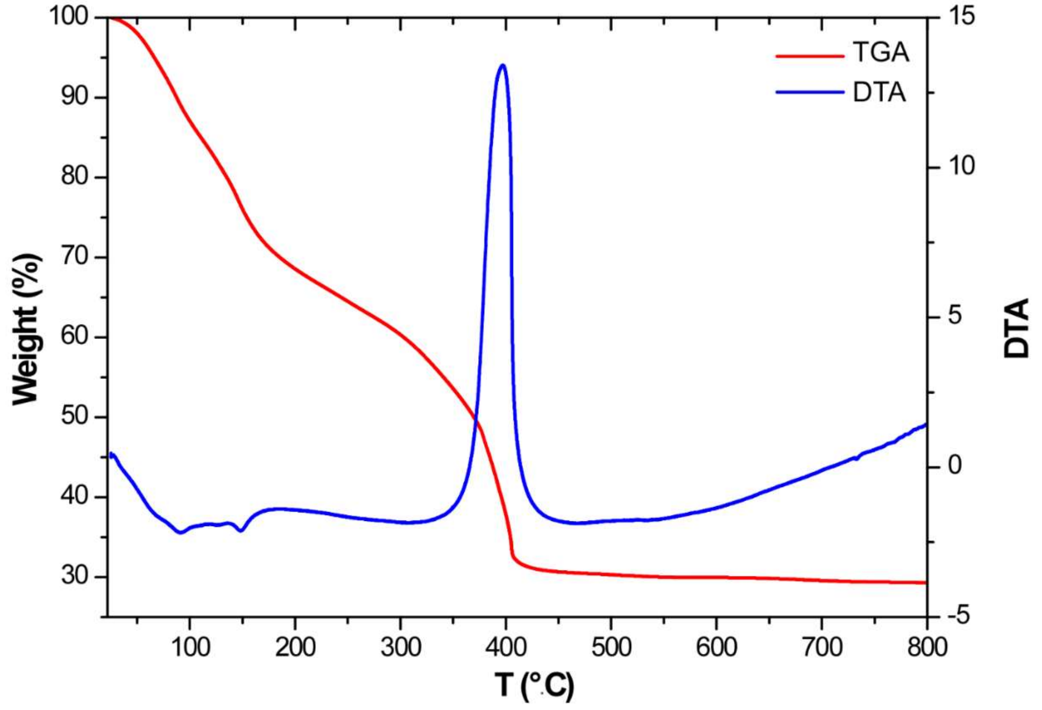

(a)

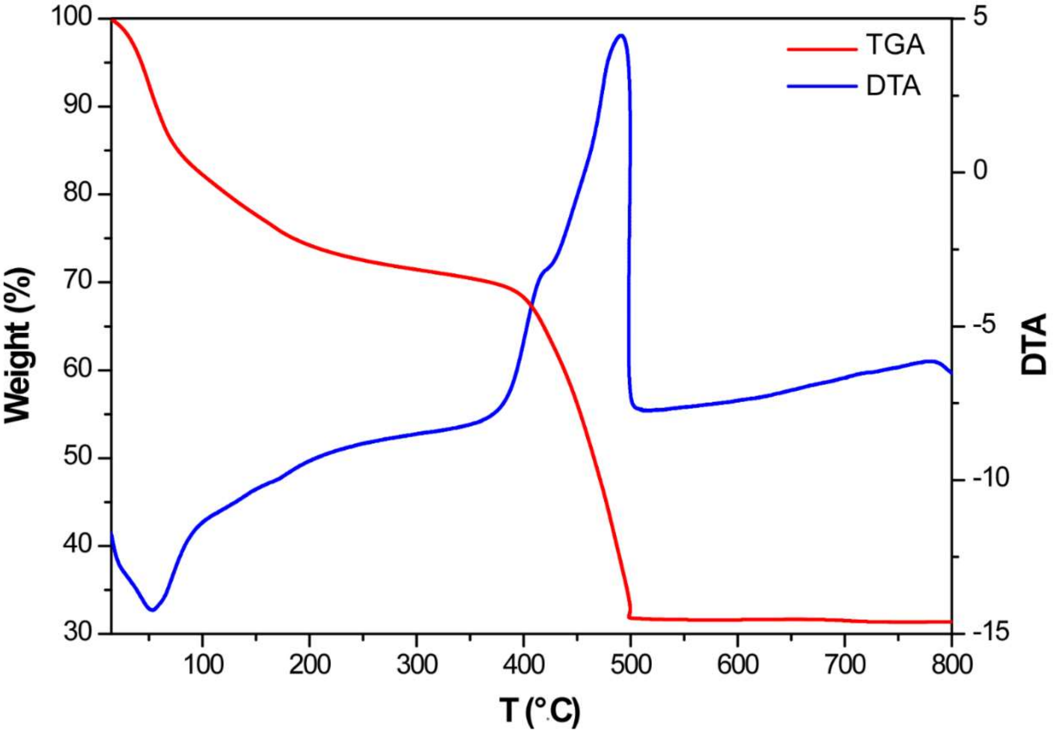

(b)

**Figure S11.** Thermogravimetric data (TGA-DTA curves) for compounds **3** (a) and **4** (b).

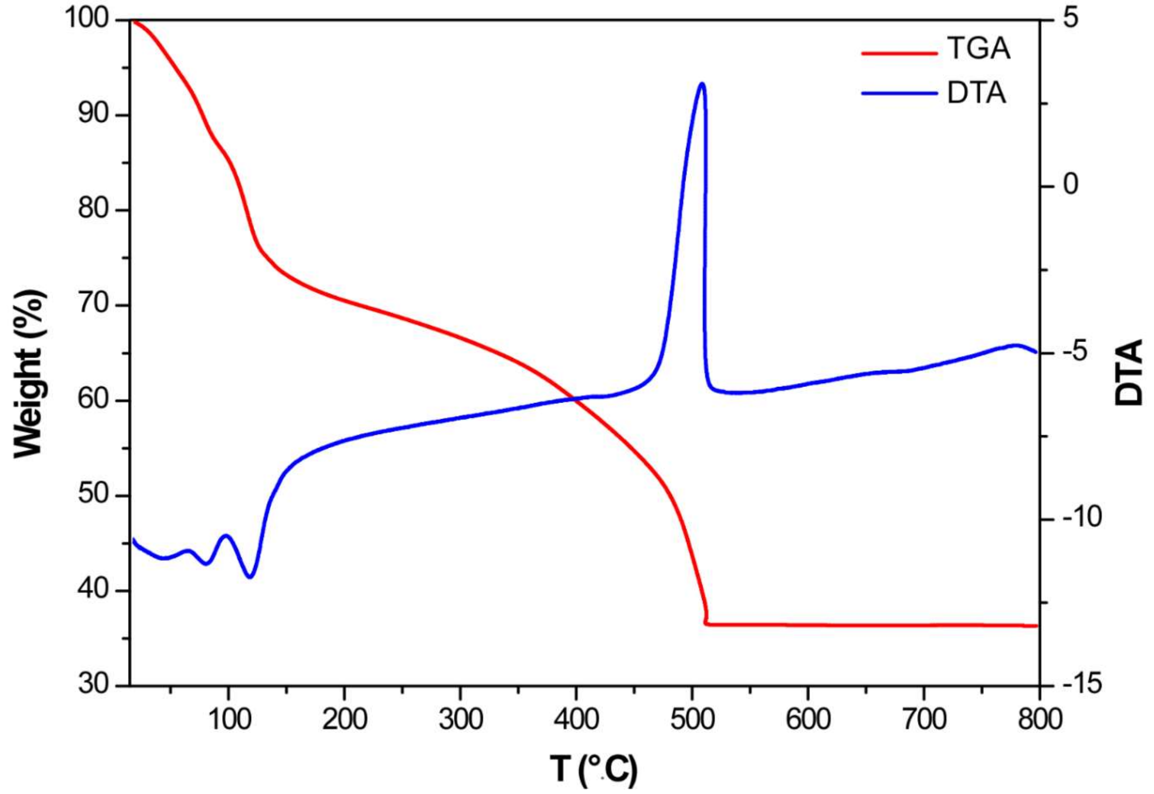


(a)

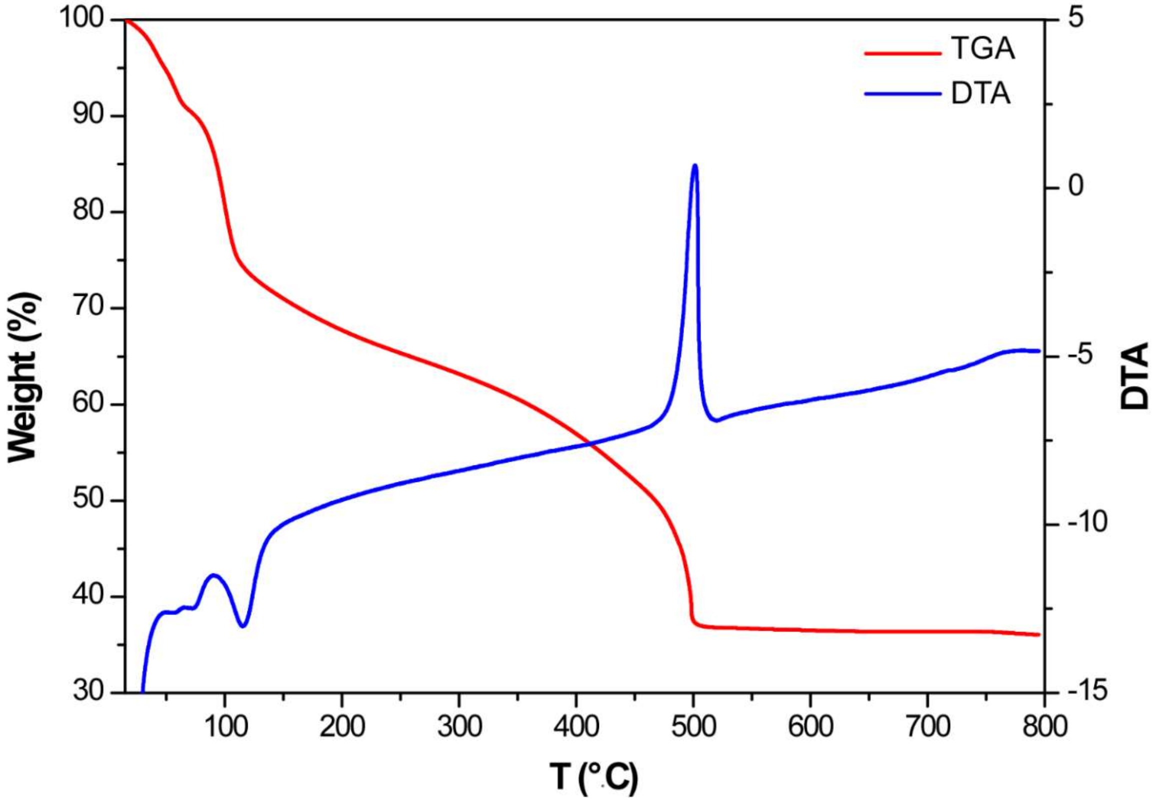


(b)

**Figure S12.** Thermogravimetric data (TGA-DTA curves) for pentameric compounds **5** (a) and **6** (b).

## S5. MASS SPECTROMETRY (MS)

To get deeper insight on the role of water for the initial nucleation process of zirconium-carboxylate systems in alcoholic media, we have carried out ESI-MS measurements for all three ligands tested (benzoic acid, and 2-hydroxy- and 3-hydroxy-benzoixc acid) at different acidic pH values (-0.5, 0.0, 0.5 and 1.0).

The experiments were run both in positive and negative ionization mode, but positive mode showed to be more informative as in the negative mode only signals belonging to the presence of organic molecules, coming probably from the ionization and subsequent fragmentation of the solvent molecules, were detected. However, the negative ionization modes provide some clues on the zirconium speciation.

The small amount of water present in the ethanol solvent is enough to allow the presence of chloride/hydroxide/oxide mixed zirconium species. It is also worth mentioning that the presence of hydroxides and oxides helps providing polynuclear entities. The behavior of the $ZrCl_4$ and benzoic acid mixture is described in detail in main text. Both pentameric and hexameric signals are present since the beginning but the relative intensity ratio is displaced towards the hexameric species upon basification.

In the case of the $ZrCl_4$ and 2-hydroxybenzoic or 3-hydroxybenzoic acids , at low pH values we find a huge noisy spectral background, with signals associated to pentameric species, together with low intensity signals in the higher *m/z* region that correspond to hexamerics entities. The m/z spacing of the signals indicates a 2+ charge for all the species found in solution. As the pH value is increased, through the dropwise addition of water, we can observe how the main signals corresponding to pentameric species decrease in intensity, the background noise disappears and the signals belonging to hexameric species begin to acquire a greater intensity, as observed in the images below. It is worth mentioning that in the case of 3-hydroxybenzoato ligand signals associated with hexameric species do not begin to have notoriety until positive pH values.

NOTE: Regarding the measured pH values, if the calibration of the electrode is performed in aqueous buffers, but the measurement is performed in a different solvent, the measured pH requires to subtract a correction constant: ${}^{s}_{s}pH = {}^{s}_{w}pH - \delta$, where ${}^{s}_{s}pH$ and ${}^{s}_{w}pH$ would in this case correspond to the pH for solvent media and measured pH, while δ is a correction constant. This constant depends of the solvent and it can be approached to –2.54 for ethanol. The pH values mentioned in this work have not been corrected and correspond to ${}^{s}_{w}pH.$ Note that the term ${}^{w}_{w}pH$ is used for the pH of an aqueous solution when the glass electrode has been calibrated with aqueous buffers. The mixed term ${}^{s}_{w}pH$ is used when the calibration is performed in aqueous buffers but the measurement is performed in a different solvent. Similarly, ${}^{s}_{s}pH$ is used if the correction constant has been subtracted from the measured ${}^{s}_{w}pH$.[1,2]

---

[1] Anderson, J. C.; Schieber, M. Crystal Growth in the System Lithium Oxide-Boron Trioxide-Ferric Oxide. *J. Phys. Chem.* **1963**, *67* (9), 1838–1840. https://doi.org/10.1021/j100803a023.

[2] Gibson, G. T. T.; Mohamed, M. F.; Neverov, A. A.; Brown, R. S. Potentiometric Titration of Metal Ions in Ethanol. *Inorg. Chem.* **2006**, *45* (19), 7891–7902. https://doi.org/10.1021/ic060517x.

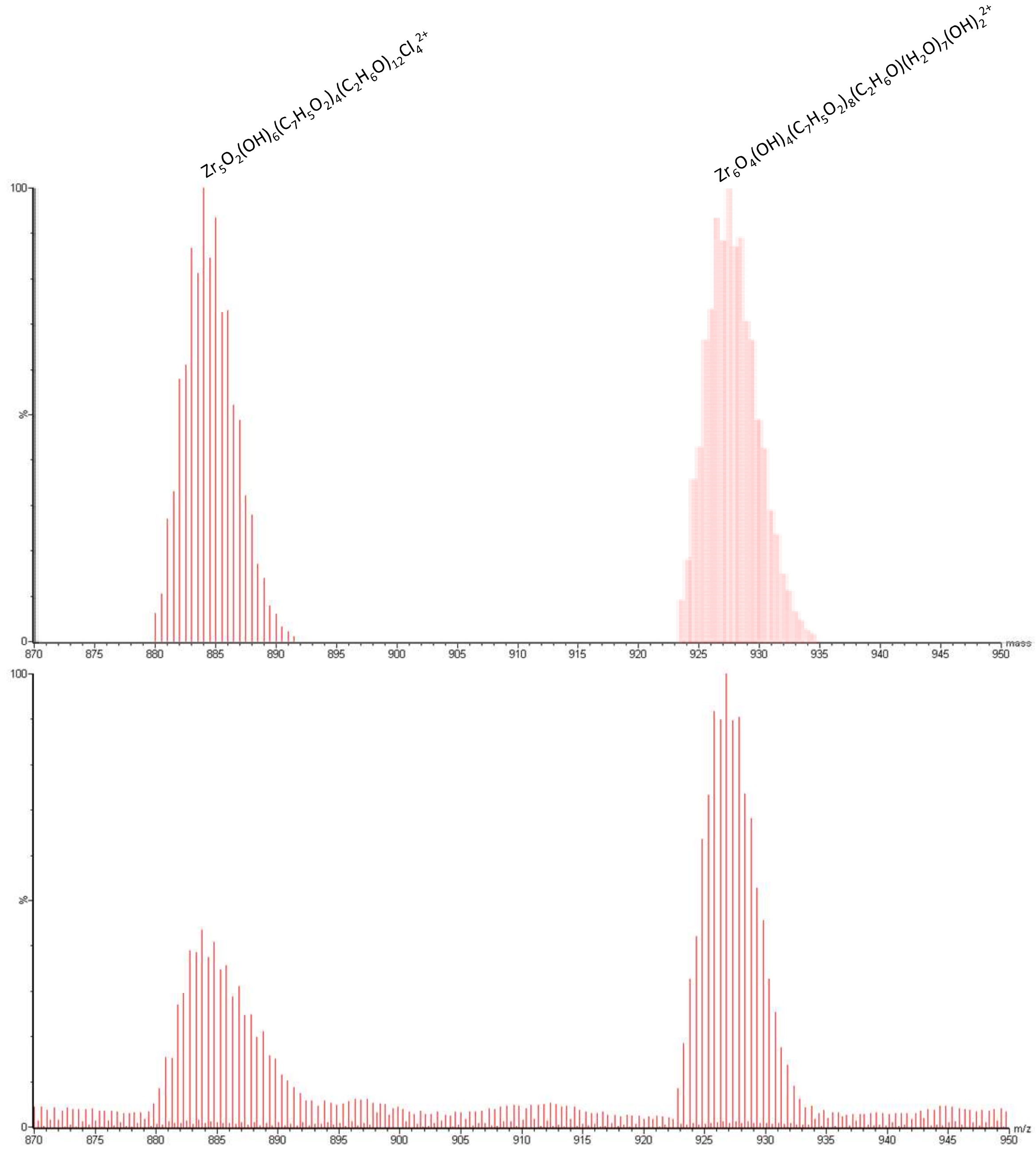


**Figure S13.** Comparison of the experimental and simulated isotopic patterns for the pentameric/hexameric species in the ESI(+)-MS spectrum of the $ZrCl_4$/benzoato system in water/ethanol mixtures at pH = +0.8.

**Table S4.** Comparison of the experimental and simulated isotopic patterns for the pentameric/hexameric species in the ESI(+)-MS spectrum of the $ZrCl_4$/benzoato system in water/ethanol mixtures at diferent pH values.

| EXPERIMENTAL | | SIMULATED | | |
|---|---|---|---|---|
| $(m/z)_{max}$ | $\Delta_{m/z}$* | $(m/z)_{max}$ | $\Delta_{m/z}$* | Formula |
| 883.84 | 881.84 – 887.34 | 884.01 | 882.01 – 887.01 | $Zr_5O_2(OH)_6(C_7H_5O_3)_4(C_2H_6O)_{12}Cl_4^{2+}$ |
| 926.86 | 924.36 – 929.87 | 926.89 | 924.39 – 929.39 | $Zr_6O_4(OH)_4(C_7H_5O_2)_8(H_2O)_7(C_2H_6O)(OH)_2^{2+}$ |

* Width at half height with respect to the maximum peak of the species isotopic distribution pattern (measured as the peak closest to that value).

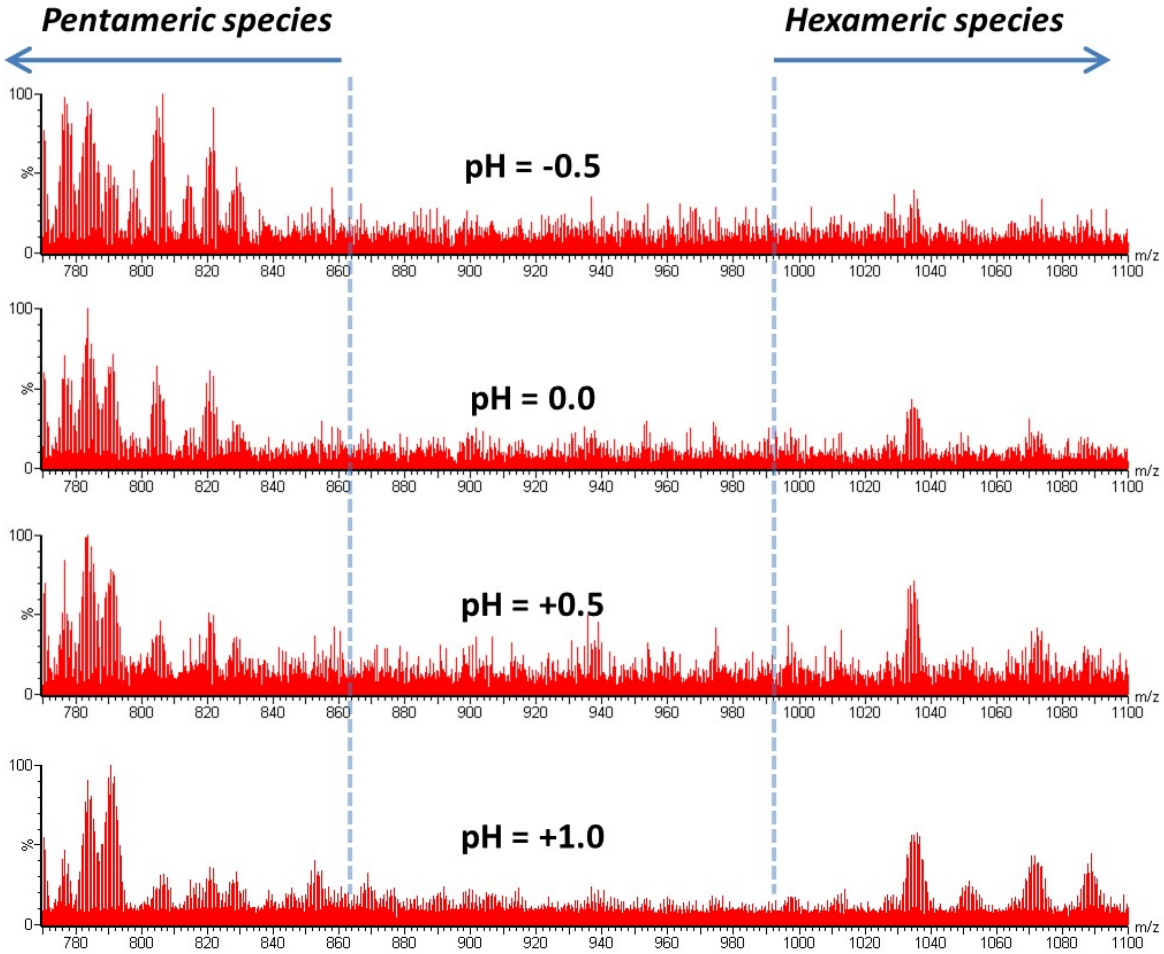


**Figure S14.** Comparison of the different mass spectra obtained for the $ZrCl_4$/2-hydroxybenzoic acid system in water/ethanol mixtures at different acidic pH values.

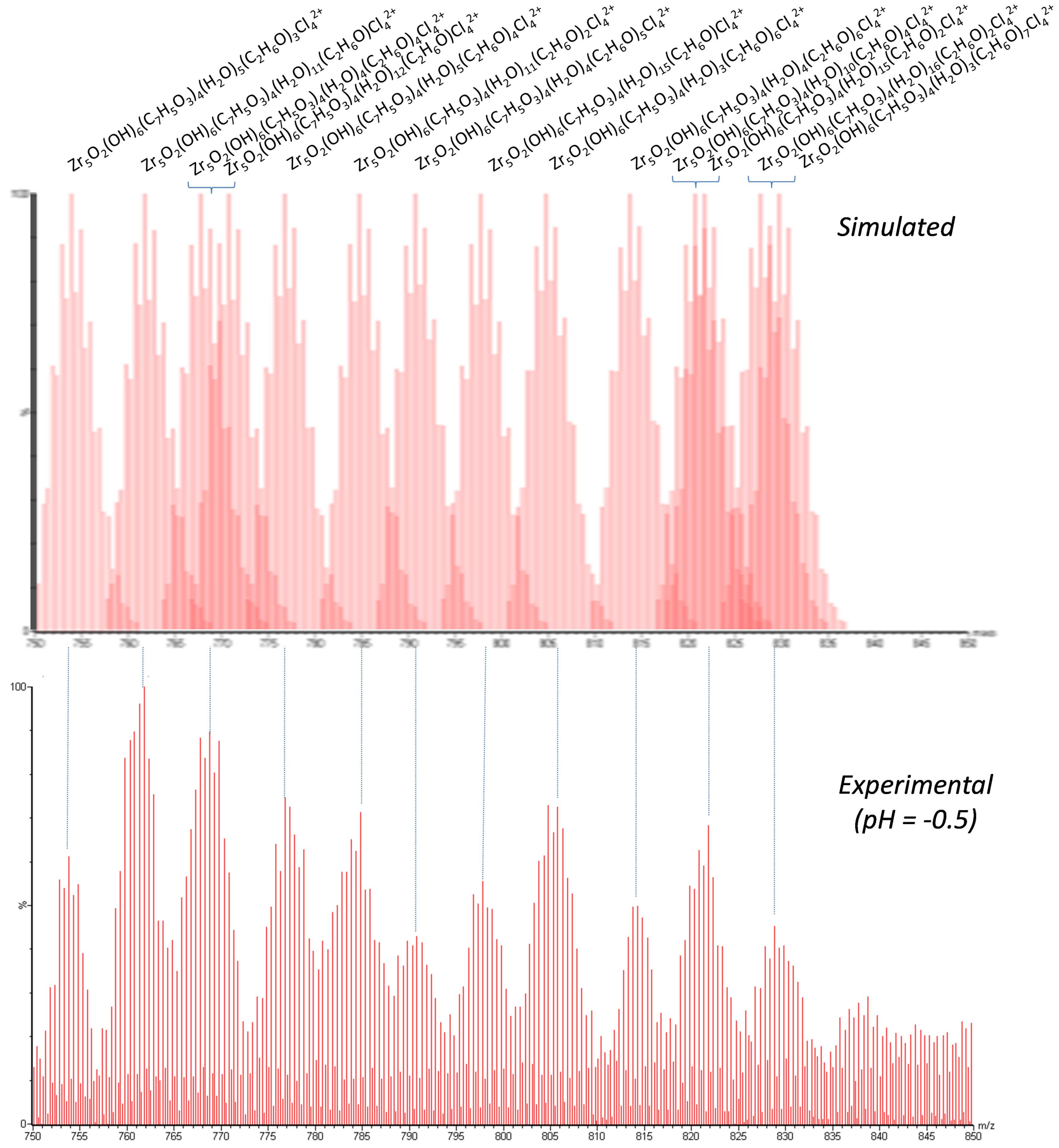


**Figure S15.** Comparison of the experimental and simulated isotopic patterns for the pentameric species in the ESI(+)-MS spectrum of the $ZrCl_4$/2-hydroxybenzoic acid system in water/ethanol mixtures at pH = –0.5.

**Table S5.** Comparison of the experimental and simulated isotopic patterns for the hexameric species in the ESI(+)-MS spectrum of the $ZrCl_4$/2-hydroxybenzoic acid system in water/ethanol mixtures at pH = -0.5

| EXPERIMENTAL | | SIMULATED | | |
|---|---|---|---|---|
| $(m/z)_{max}$ | $\Delta_{m/z}$* | $(m/z)_{max}$ | $\Delta_{m/z}$* | Formula |
| 753.79 | 751.80 – 755.79 | 753.84 | 751.84 – 755.84 | $Zr_5O_2(OH)_6(C_7H_5O_3)_4(H_2O)_5(C_2H_6O)_3Cl_4^{2+}$ |
| 761.79 | 758.79 – 763.80 | 761.83 | 759.83 – 763.82 | $Zr_5O_2(OH)_6(C_7H_5O_3)_4(H_2O)_{11}(C_2H_6O)Cl_4^{2+}$ |
| 768.80 | 765.81 – 770.80 | 767.86 | 765.86 – 769.86 | $Zr_5O_2(OH)_6(C_7H_5O_3)_4(H_2O)_4(C_2H_6O)_4Cl_4^{2+}$ |
| | | 770.83 | 768.83 – 772.83 | $Zr_5O_2(OH)_6(C_7H_5O_3)_4(H_2O)_{12}(C_2H_6O)Cl_4^{2+}$ |
| 776.81 | 774.82 – 779.82 | 776.86 | 774.86 – 779.86 | $Zr_5O_2(OH)_6(C_7H_5O_3)_4(H_2O)_5(C_2H_6O)_4Cl_4^{2+}$ |
| 784.82 | 780.82 – 786.82 | 784.85 | 780.32 – 787.82 | $Zr_5O_2(OH)_6(C_7H_5O_3)_4(H_2O)_{11}(C_2H_6O)_2Cl_4^{2+}$ |
| 790.83 | overlaping – 792.83 | 790.88 | 788.88 – 793.88 | $Zr_5O_2(OH)_6(C_7H_5O_3)_4(H_2O)_4(C_2H_6O)_5Cl_4^{2+}$ |
| 797.78 | 795.77 – 800.79 | 797.85 | 795.85 – 800.85 | $Zr_5O_2(OH)_6(C_7H_5O_3)_4(H_2O)_{15}(C_2H_6O)Cl_4^{2+}$ |
| 804.79 | 802.79 – 807.79 | 804.89 | 802.89 – 807.89 | $Zr_5O_2(OH)_6(C_7H_5O_3)_4(H_2O)_3(C_2H_6O)_6Cl_4^{2+}$ |
| 813.79 | 811.79 – 816.75 | 813.90 | 811.90 – 816.90 | $Zr_5O_2(OH)_6(C_7H_5O_3)_4(H_2O)_4(C_2H_6O)_6Cl_4^{2+}$ |
| 820.81/821.81 | 818.80 – 823.30 | 820.87 | 818.87 – 823.87 | $Zr_5O_2(OH)_6(C_7H_5O_3)_4(H_2O)_{15}(C_2H_6O)_2Cl_4^{2+}$ |
| | | 821.89 | 819.89 – 824.89 | $Zr_5O_2(OH)_6(C_7H_5O_3)_4(H_2O)_{10}(C_2H_6O)_4Cl_4^{2+}$ |
| 828.82 | 825.82 – 831.31 | 827.91 | 825.91 – 830.91 | $Zr_5O_2(OH)_6(C_7H_5O_3)_4(H_2O)_3(C_2H_6O)_7Cl_4^{2+}$ |
| | | 829.88 | 827.88 – 832.88 | $Zr_5O_2(OH)_6(C_7H_5O_3)_4(H_2O)_{16}(C_2H_6O)_2Cl_4^{2+}$ |

* Width at half height with respect to the maximum peak of the species isotopic distribution pattern.

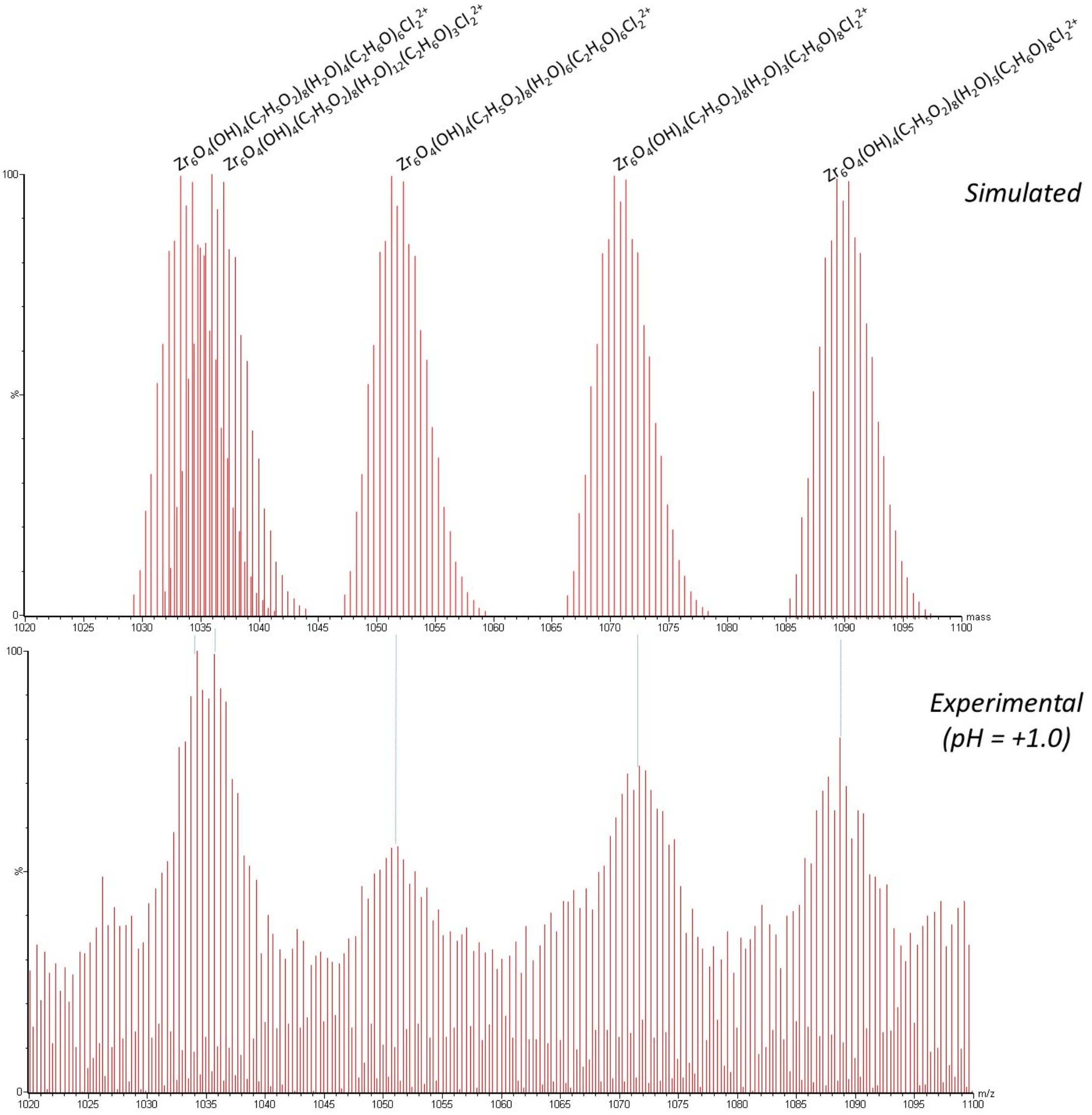


**Figure S16.** Comparison of the experimental and simulated isotopic patterns for the hexameric species in the ESI(+)-MS spectrum of the $ZrCl_4$/2-hydroxybenzoic acid system in water/ethanol mixtures at pH = 1.

**Table S6.** Comparison of the experimental and simulated isotopic patterns for the hexameric species in the ESI(+)-MS spectrum of the $ZrCl_4$/2-hydroxybenzoic acid system in water/ethanol mixtures at pH = 1.0

| EXPERIMENTAL | | SIMULATED | | |
|---|---|---|---|---|
| $(m/z)_{max}$ | $\Delta_{m/z}$* | $(m/z)_{max}$ | $\Delta_{m/z}$* | Formula |
| 1035.71 | 1030.72 – 1037.71 | 1032.86 | 1030.86 – 1035.86 | $Zr_6O_4(OH)_4(C_7H_5O_3)_8(H_2O)_2(C_2H_6O)_4Cl_2^{2+}$ |
| | | 1035.84 | 1033.84 – 1038.84 | $Zr_6O_4(OH)_4(C_7H_5O_3)_8(H_2O)_9(C_2H_6O)_2Cl_2^{2+}$ |
| 1051.22 | 1048.23 – 1055.63 | 1049.86 | 1047.86 – 1052.86 | $Zr_6O_4(OH)_4(C_7H_5O_3)_8(H_2O)_{10}(C_2H_6O)Cl_2^{2+}$ |
| | | 1050.88 | 1048.88 – 1053.88 | $Zr_6O_4(OH)_4(C_7H_5O_3)_8(H_2O)_4(C_2H_6O)_4Cl_2^{2+}$ |
| 1071.71 | 1068.21 – 1074.66 | 1071.86 | 1069.86 – 1074.87 | $Zr_6O_4(OH)_4(C_7H_5O_3)_8(H_2O)_{14}(C_2H_6O)Cl_2^{2+}$ |
| 1087.72 / 1088.71 | 1085.72 – 1090.67 | 1087.91 / 1088.92 | 1085.92 – 1090.91 | $Zr_6O_4(OH)_4(C_7H_5O_3)_8(H_2O)_3(C_2H_6O)_6Cl_2^{2+}$ |

* Width at half height with respect to the maximum peak of the species isotopic distribution pattern.

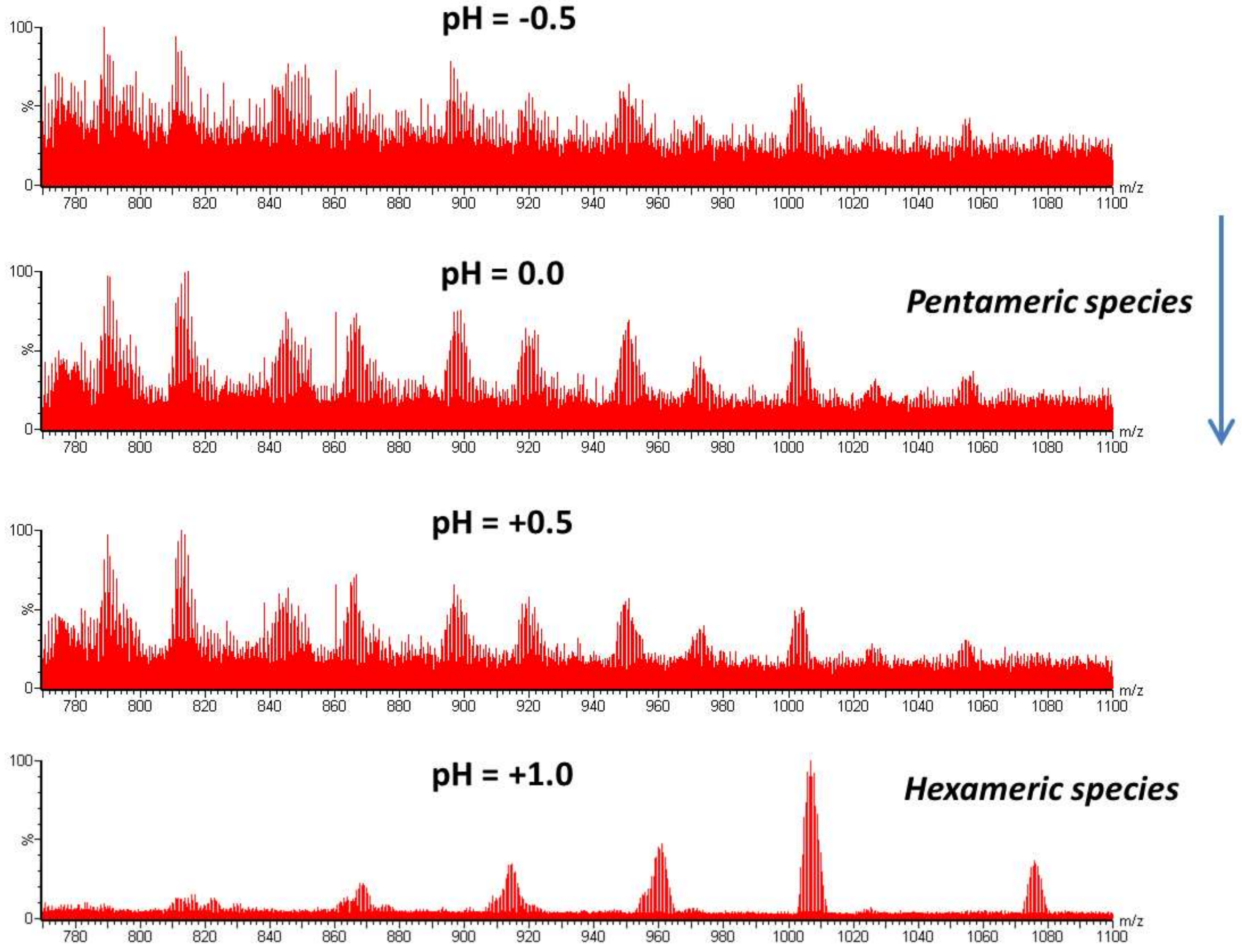


**Figure S17.** Comparison of the different mass spectra obtained for the $ZrCl_4$/3-hydroxybenzoic acid system in water/ethanol mixtures at different very acidic pH values.

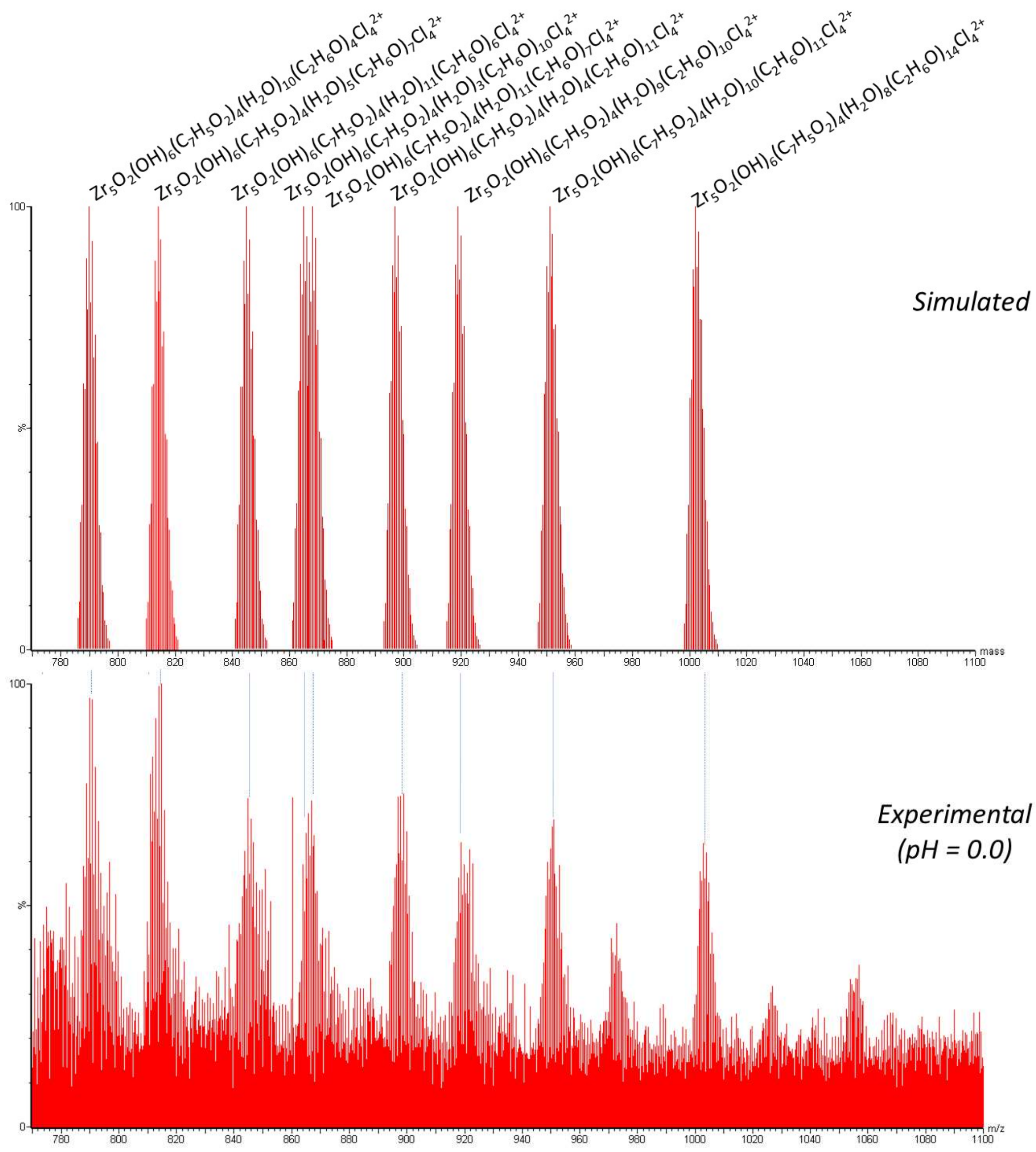


**Figure S18.** Comparison of the experimental and simulated isotopic patterns for the pentameric species in the ESI(+)-MS spectrum of the $ZrCl_4$/3-hydroxybenzoic acid system in water/ethanol mixtures at pH = 0.0.

**Table S7.** Comparison of the experimental and simulated isotopic patterns for the hexameric species in the ESI(+)-MS spectrum of the $ZrCl_4$/3-hydroxybenzoic acid system in water/ethanol mixtures at pH = 0.0

| EXPERIMENTAL | | SIMULATED | | |
|---|---|---|---|---|
| **$(m/z)_{max}$** | **$\Delta_{m/z}$*** | **$(m/z)_{max}$** | **$\Delta_{m/z}$*** | **Formula** |
| 790.83 | 787.83 – 794.82 | 790.87 | 788.37 – 793.37 | $Zr_5O_2(OH)_6(C_7H_5O_3)_4(H_2O)_4(C_2H_6O)_5Cl_4^{2+}$ |
| 813.85 | 810.87 – 816.82 | 813.89 | 811.39 – 816.39 | $Zr_5O_2(OH)_6(C_7H_5O_3)_4(H_2O)_4(C_2H_6O)_6Cl_4^{2+}$ |
| 844.82 | 841.84 – 847.83 | 844.91 | 842.90 – 847.40 | $Zr_5O_2(OH)_6(C_7H_5O_3)_4(H_2O)_{10}(C_2H_6O)_5Cl_4^{2+}$ |
| 866.86 | 863.39 – 869.33 | 866.91 | 864.91 – 869.91 | $Zr_5O_2(OH)_6(C_7H_5O_3)_4(H_2O)_{15}(C_2H_6O)_4Cl_4^{2+}$ |
| 897.83 | 894.34 – 899.82 | 897.92 | 894.47 – 899.97 | $Zr_5O_2(OH)_6(C_7H_5O_3)_4(H_2O)_{21}(C_2H_6O)_3Cl_4^{2+}$ |
| 918.85 | 916.38 – 924.81 | 918.98 | 916.98 – 921.48 | $Zr_5O_2(OH)_6(C_7H_5O_3)_4(H_2O)_8(C_2H_6O)_9Cl_4^{2+}$ |
| 950.83 | 946.86 – 953.81 | 951.01 | 949.01 – 954.01 | $Zr_5O_2(OH)_6(C_7H_5O_3)_4(H_2O)_9(C_2H_6O)_{10}Cl_4^{2+}$ |
| 972.86 | 969.88 – 976.81 | 973.01 | 970.01 – 975.51 | $Zr_5O_2(OH)_6(C_7H_5O_3)_4(H_2O)_{14}(C_2H_6O)_9Cl_4^{2+}$ |
| 1002.85 | 1000.37 – 1006.83 | 1003.07 | 1001.07 – 1006.07 | $Zr_5O_2(OH)_6(C_7H_5O_3)_4(H_2O)_2(C_2H_6O)_{15}Cl_4^{2+}$ |

* Width at half height with respect to the maximum peak of the species isotopic distribution pattern.

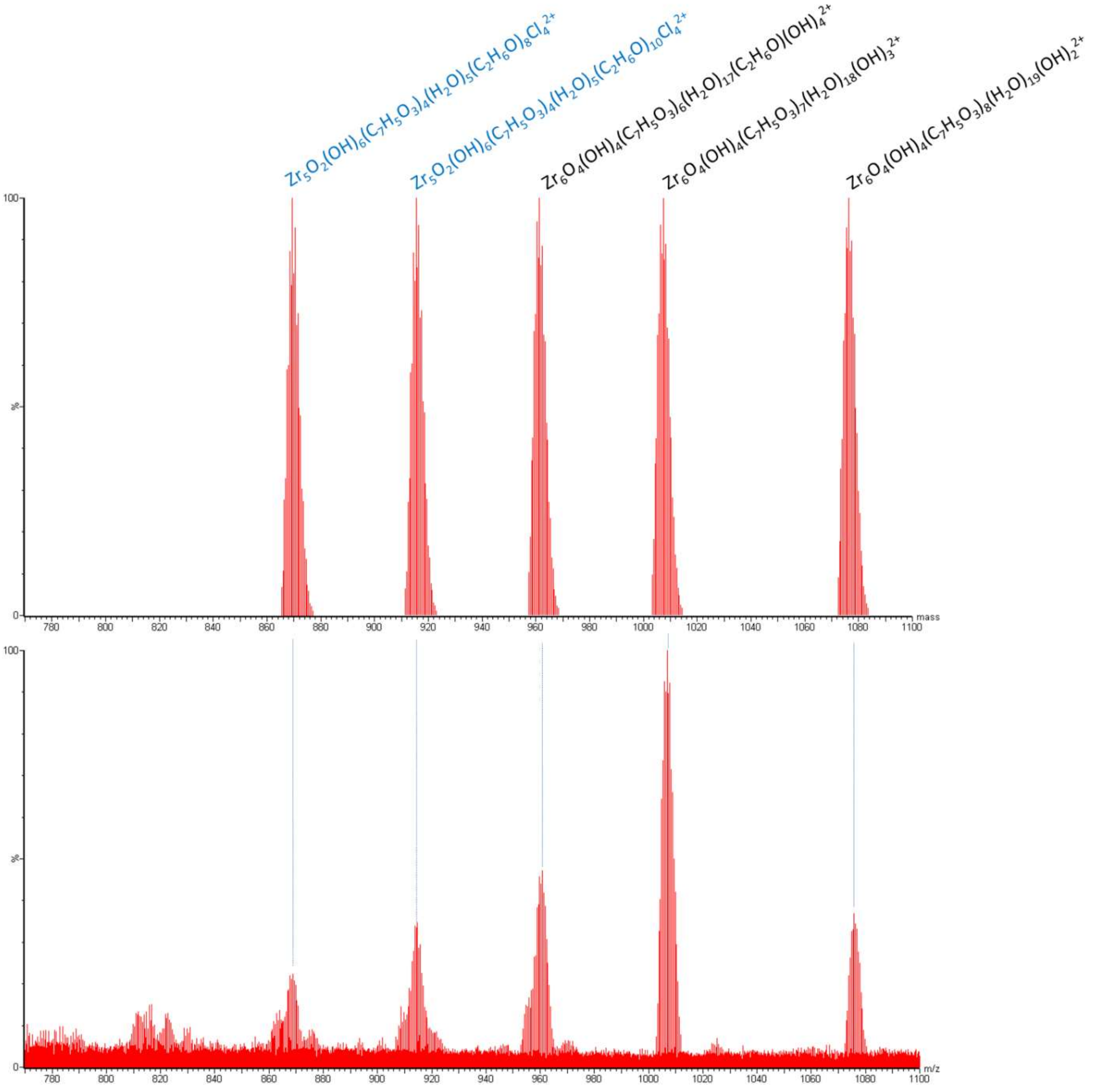


**Figure S19.** Comparison of the experimental and simulated isotopic patterns for the hexameric species in the ESI(+)-MS spectrum of the $ZrCl_4$/3-hydroxybenzoic acid system in water/ethanol mixtures at pH = 1.0

**Table S8.** Comparison of the experimental and simulated isotopic patterns for the hexameric species in the ESI(+)-MS spectrum of the $ZrCl_4$/3-hydroxybenzoic acid system in water/ethanol mixtures at pH = 1.0

| **EXPERIMENTAL** | | **SIMULATED** | | |
|---|---|---|---|---|
| **$(m/z)_{max}$** | **$\Delta_{m/z}$*** | **$(m/z)_{max}$** | **$\Delta_{m/z}$*** | **Formula** |
| 868.78 | 866.27 – 870.78 | 868.94 | 866.94 – 871.44 | $Zr_5O_2(OH)_6(C_7H_5O_3)_4(H_2O)_5(C_2H_6O)_8Cl_4^{2+}$ |
| 914.79 | 911.79 – 916.79 | 914.98 | 912.98 – 917.48 | $Zr_5O_2(OH)_6(C_7H_5O_3)_4(H_2O)_5(C_2H_6O)_{10}Cl_4^{2+}$ |
| 960.81 | 958.30 – 962.81 | 960.90 | 958.40 – 963.40 | $Zr_6O_4(OH)_4(C_7H_5O_3)_6(H_2O)_{17}(C_2H_6O)(OH)_4^{2+}$ |
| 1006.83 | 1004.33 – 1009.33 | 1006.89 | 1004.39 – 1009.39 | $Zr_6O_4(OH)_4(C_7H_5O_3)_7(H_2O)_{18}(OH)_3^{2+}$ |
| 1075.84 | 1073.34 – 1078.34 | 1075.91 | 1073.41 – 1078.41 | $Zr_6O_4(OH)_4(C_7H_5O_3)_8(H_2O)_{19}(OH)_2^{2+}$ |

* Width at half height with respect to the maximum peak of the species isotopic distribution pattern.

## S6. SOLID STATE NUCLEAR MAGNETIC RESONANCE (NMR)

Solid state magic angle spinning (MAS) $^{1}H$- and $^{13}C$-NMR measurements have been carried out in order to be able to differentiate and corroborate that the cluster of the unsaturated entities remain stable after removing them from their mother liquors. Solid state measurements did not allow us to integrate the NMR signals to see how many atoms they correspond to. However, it allows us to prove the presence of benzenecarboxylate ligand in **1**, **2**, **5** and **6** and the corresponding hydroxylated benzenecarboxylate in **3** and **4.**

Compounds **1** and **2**: $^{13}$C-NMR spectra (Figure S20) show two different signals at 172 ppm attributable to the carbon of the carboxylate group and another one, at 130 ppm, with a higher intensity, assigned to the aromatic carbons. In the case of $^{1}$H-NMR two different signals are also observed. The first one at a value around 3.6 ppm is associated to the hydrogen atoms of the water molecules of the cluster (blue). The second one, around 7.2 ppm is related to the hydrogen atoms of aromatic ring.

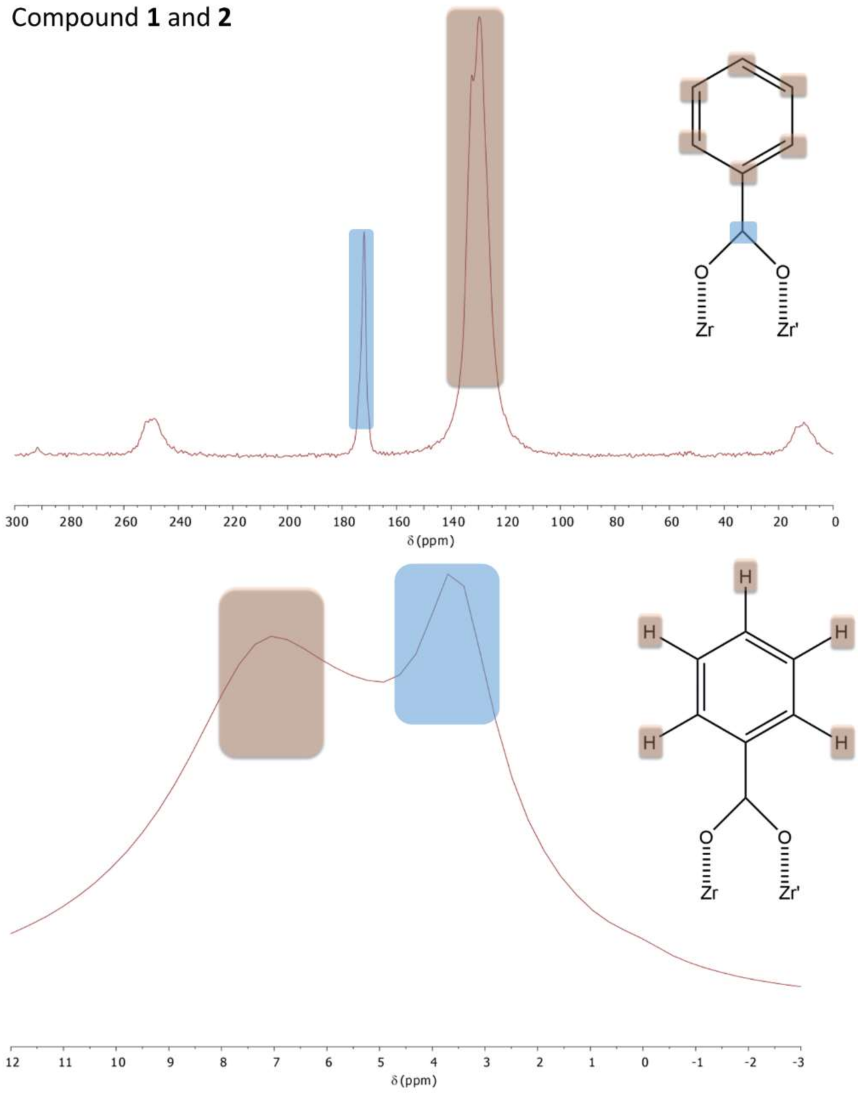


**Figure S20.** $^{13}$C-NMR (upper) and $^{1}$H-NMR (bottom) MAS NMR spectra for bulk samples of compounds **1** and **2.**

Compound **3**: $^{13}$C-NMR spectra shows four different signals (Figure S21), the first one around 173 ppm attributable to the carbon of the carboxilato group, the second one around 160 ppm attributable to the carbon of the hydroxide group, the last two ones around 137 and 113 ppm belong to the remaining aromatic carbons. In the case of $^{1}$H-NMR three different signals are also observed. The first one at a value around 2.9 ppm is associated to the water molecules of the cluster. The second one, around 6.5 ppm is due the aromatic hydrogen atoms (blue). In addition, a signal located at 9.6 ppm is attributable the hydroxyl group.

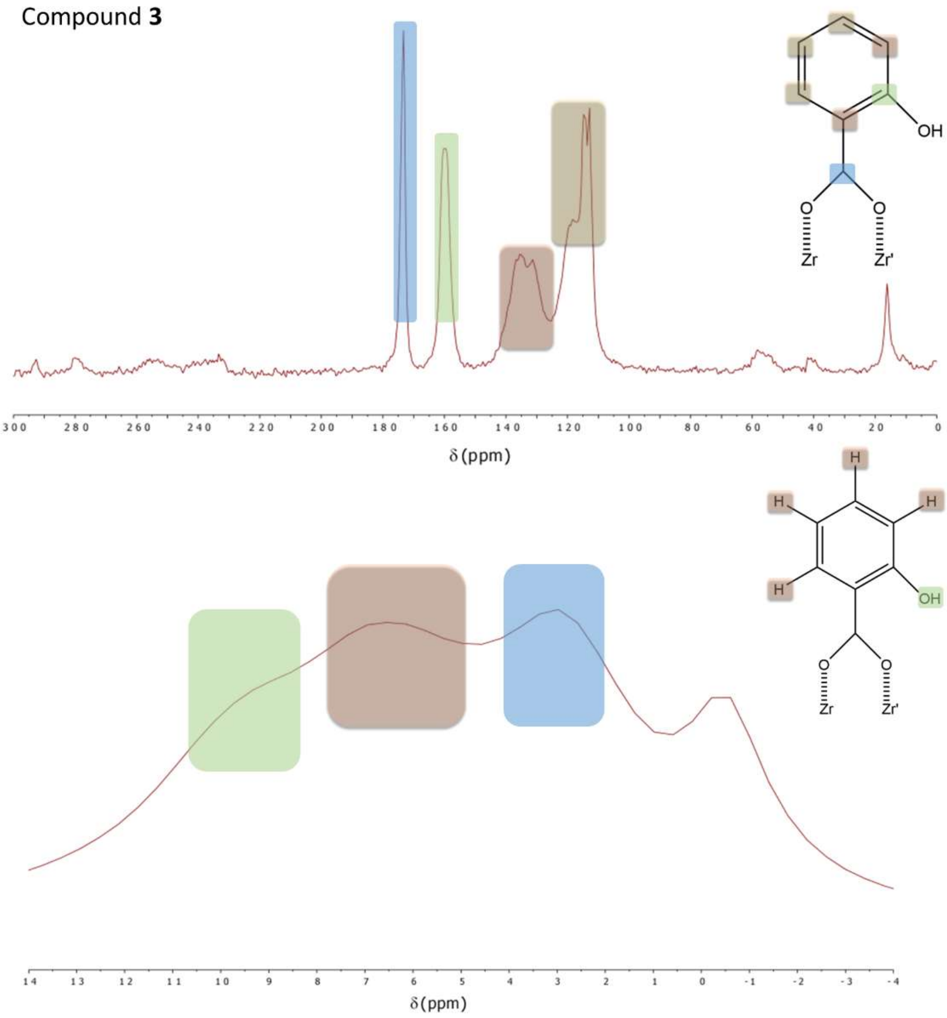


**Figure S21.** $^{13}$C-NMR (upper) and $^{1}$H-NMR (bottom) MAS NMR spectra for **3**.

Compound **4**: in the spectra of $^{13}C$-NMR four different signals are observed (Figure S22), the first one around 171 ppm attributable to the carbon of the carboxilato group, the second one around 153 ppm attributable to the carbon of the hydroxide group, the last two ones around 131-128 and 121-115 ppm belong to the remaining aromatic carbons. In the case of $^{1}H$-NMR only two different signals are clearly observed. The first one at a value around 4.3 ppm is associated to the water molecules of the cluster. The second one, around 7.1 ppm is due the aromatic hydrogen atoms (blue).

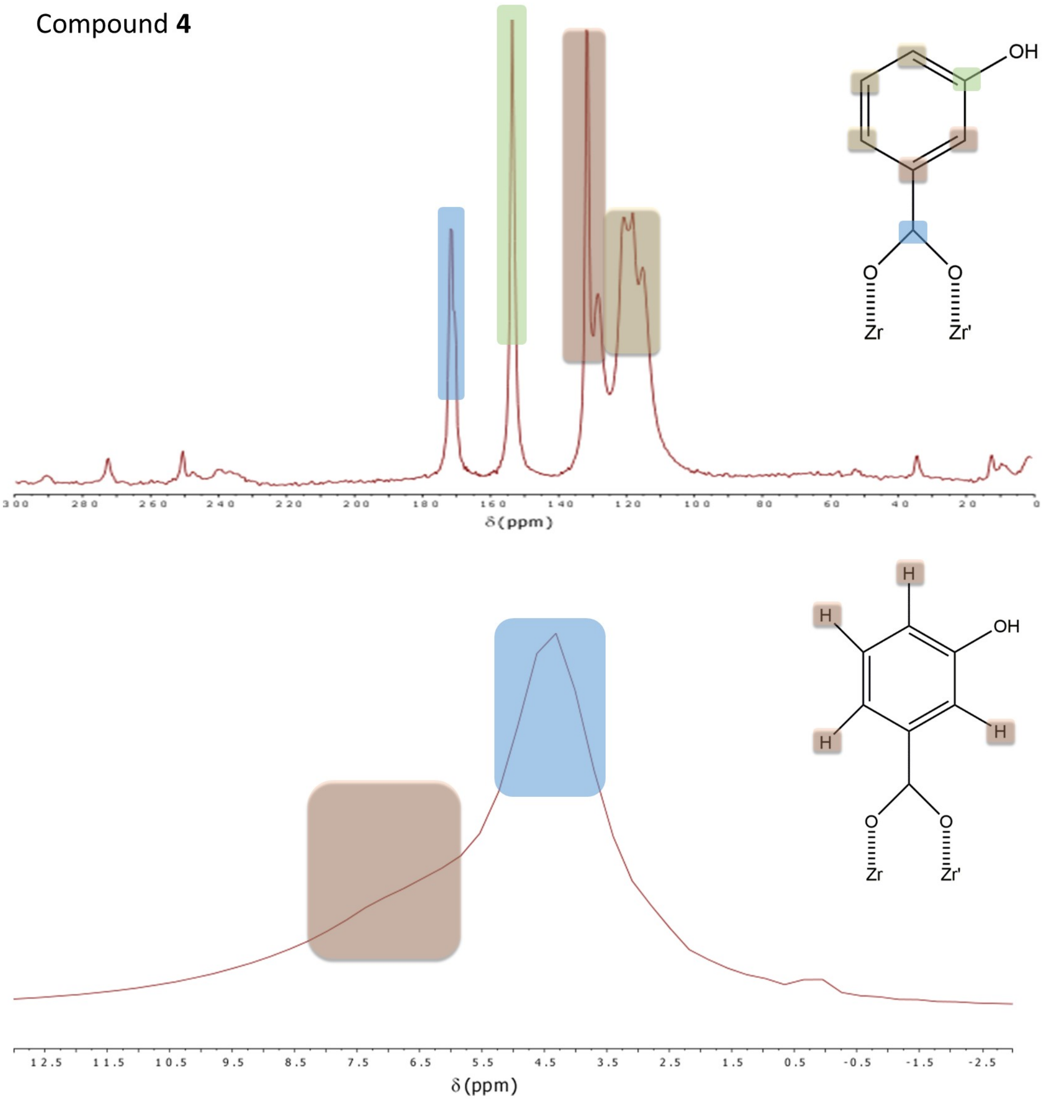


**Figure S22.** $^{13}C$-NMR (upper) and $^{1}H$-NMR (bottom) MAS NMR spectra for **4.**

Compound **5**: in the spectra of $^{13}C$-NMR four different signals are observed (Figure S23), the first one around 172 ppm attributable to the carbon of the carboxilato group, the second one around 130 ppm attributable to the aromatic carbons, the last two signals belong to the carbon atoms of the ethanol molecule attached to the cluster appearing around 58 ($CH_2$) and 17 ($CH_3$) ppm, respectively. In the case of $^{1}H$-NMR a broad band can be separated into three different contributions: the first one at a value around 7.0 ppm attributable to the aromatic hydrogen atoms, a second one around 4.3 ppm associated to the water molecules of the cluster, the third one around 3.0 ppm attributable to the $CH_2$ group of the ethanol molecule of the pentameric entity. A separated fourth signal around 0.4 ppm associated to the $CH_3$ group of the ethanol molecule is also visible.

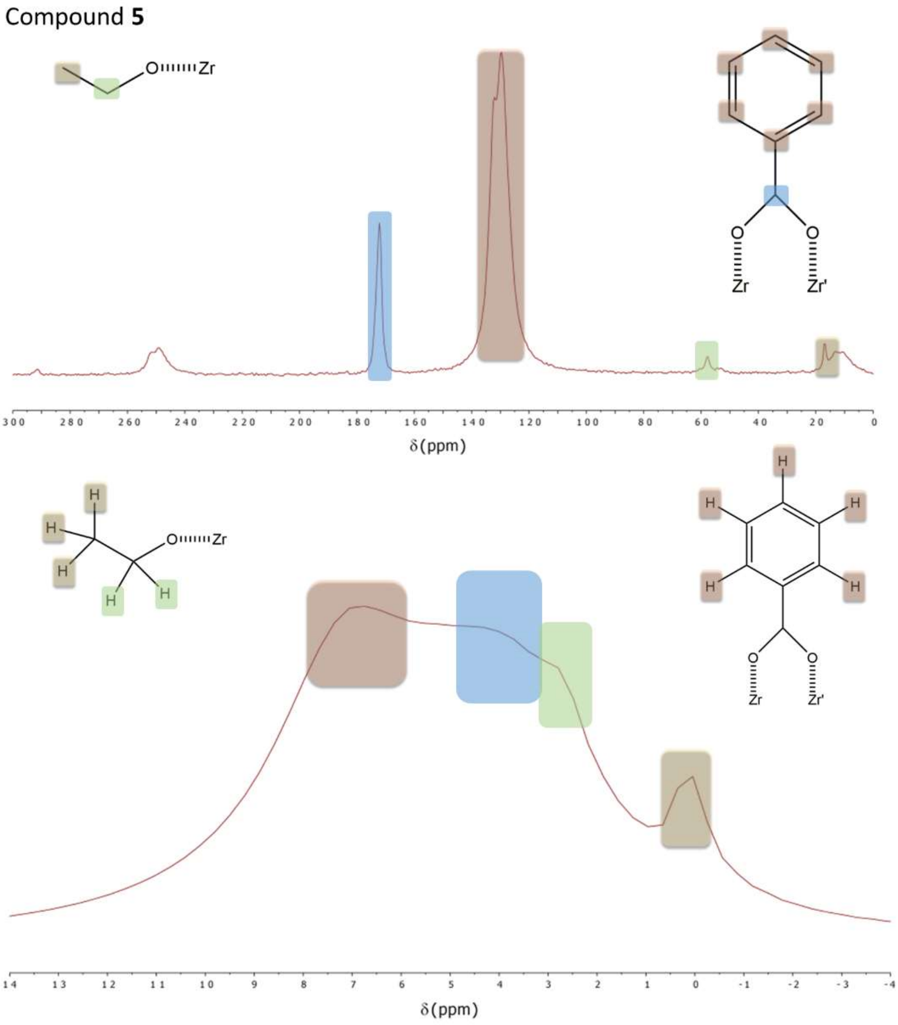


**Figure S23.** $^{13}C$-NMR (upper) and $^{1}H$-NMR (bottom) MAS NMR spectra for **5.**

Compound **6**: in the spectra of $^{13}C$-NMR four different signals are observed (Figure S24), the first one around 172 ppm attributable to the carbon of the carboxilato group, the second one around 131 ppm attributable to the aromatic carbons, the last two signals belong to the carbon atoms of the ethanol molecule attached to the cluster appearing around 63 ($CH_2$) and 22 ($CH_3$) ppm, respectively. In the case of $^{1}H$-NMR a broad band can be separated into three different contributions: the first one at a value around 7.3 ppm attributable to the aromatic hydrogen atoms, a second one around 4.5 ppm associated to the water molecules of the cluster, the third one around 2.5 ppm attributable to the $CH_2$ groups of the propanol molecule of the pentameric entity. A separated fourth signal around -0.5 ppm associated to the $CH_3$ group of the propanol molecule is also visible.

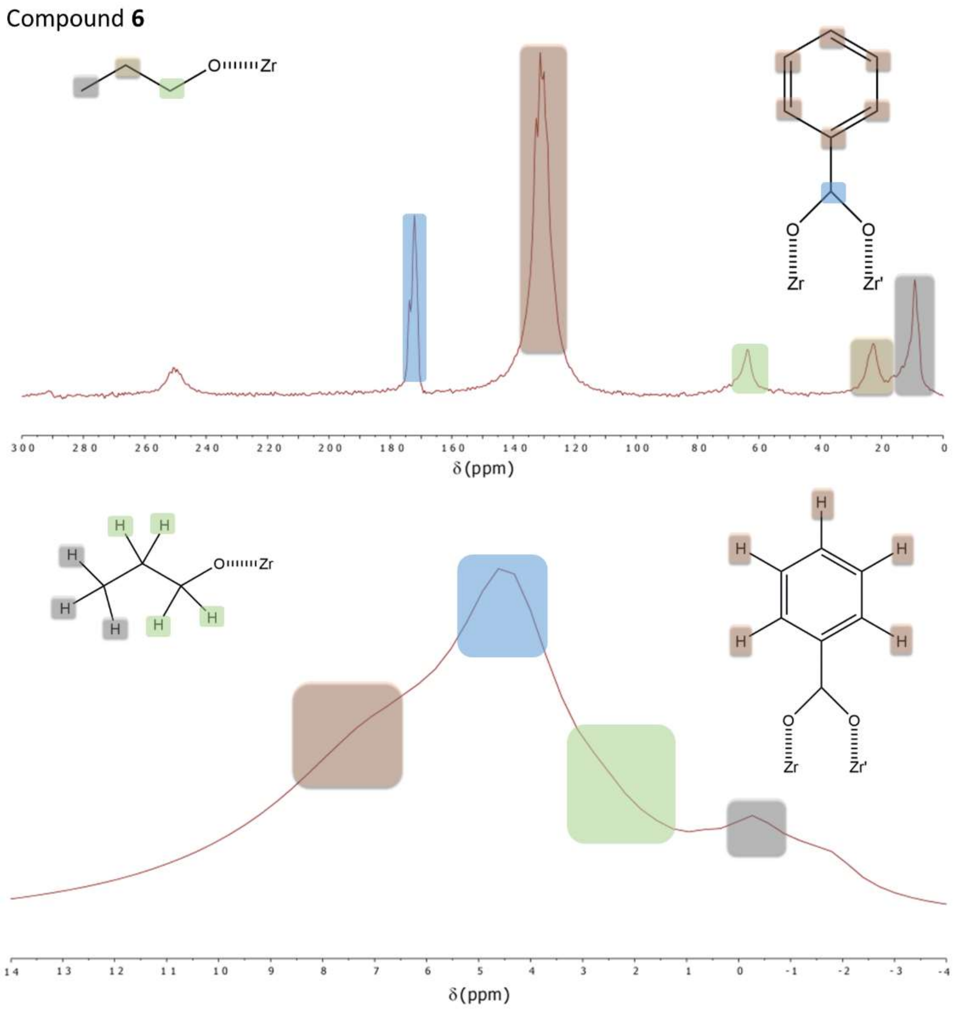


**Figure S24.** $^{13}C$-NMR (upper) and $^{1}H$-NMR (bottom) MAS NMR spectra for **6**.

## S7. REFERENCES

1. Brese, N.E.; O'Keeffe, M. *Acta Cryst.* **1991**, *B47*, 192-197.
2. Brown, I.D. *The Chemical Bond in Inorganic Chemistry: The Bond Valence Model*. Oxford University Press, 2002.
3. Trivedi, M. K.; Branton, A.; Trivedi, D.; Shettigar, H.; Bairwa, K.; Jana, S. *Nat Prod Chem Res*, **2015**, *3*, 1000186..
4. Atzori, C.; Shearer, G.C.; Maschio, L.; Civalleri, B.; Bonino,F.; Lamberti, C.; Svelle, S.; Lillerud, K. P.; Bordiga, S. *J. Phys. Chem. C* **2017**, 121, 9312–9324.
